# Sonic Hedgehog Is a Determinant of γδ T-Cell Differentiation in the Thymus

**DOI:** 10.3389/fimmu.2019.01629

**Published:** 2019-07-19

**Authors:** Konstantinos Mengrelis, Ching-In Lau, Jasmine Rowell, Anisha Solanki, Sonia Norris, Susan Ross, Masahiro Ono, Susan Outram, Tessa Crompton

**Affiliations:** ^1^UCL Great Ormond Street Institute of Child Health, London, United Kingdom; ^2^Department of Life Sciences, Imperial College London, London, United Kingdom; ^3^Department of Natural Sciences, Middlesex University, London, United Kingdom

**Keywords:** Shh, γδ T-cell, thymus, γδNKT, Smoothened, Hedgehog

## Abstract

Here we investigate the function of Hedgehog (Hh) signaling in thymic γδ T-cell maturation and subset differentiation. Analysis of Hh mutants showed that Hh signaling promotes γδ T-cell development in the thymus and influences γδ T-cell effector subset distribution. Hh-mediated transcription in thymic γδ cells increased γδ T-cell number, and promoted their maturation and increased the γδNKT subset, whereas inhibition of Hh-mediated transcription reduced the thymic γδ T-cell population and increased expression of many genes that are normally down-regulated during γδ T-cell maturation. These changes were also evident in spleen, where increased Hh signaling increased γδNKT cells, but reduced CD27-CD44+ and Vγ2+ populations. Systemic *in vivo* pharmacological Smoothened-inhibition reduced γδ T-cell and γδNKT cells in the thymus, and also reduced splenic γδ T-cell and γδNKT populations, indicating that Hh signaling also influences homeostasis of peripheral γδ T-cell populations. Taken together our data indicate that Sonic Hedgehog is an important determinant of γδ T-cell effector subset differentiation.

## Introduction

Gamma delta (γδ) T-cells are a conserved population of lymphocytes, which like αβ T-cells develop in the thymus. αβ T-cell development and γδ T-cell development diverge at the CD44+CD25+CD4-CD8- (double negative [DN2]) stage of thymocyte development, during which rearrangement of β-, γ-, and δ-chains of the TCR is initiated. Strength of TCR signal is believed to influence the αβ/γδ lineage choice, with stronger signaling leading to γδ T-cell commitment, and weaker signaling leading to differentiation along the αβ T-cell lineage (1–3). Thus, successful rearrangement of γ- and δ-chains, and functional expression and signaling though the γδTCR complex drives differentiation into the γδ T-cell lineage, supported by expression of the transcription factor Sox13. Cells that have not produced a functional γδTCR, however, undergo β-selection for differentiation along the αβ T-cell lineage (4, 5).

γδ T-cells first develop before αβ T-cells during ontogeny, with waves of development leading to distinct subsets of γδ T-cells which home to particular anatomical sites, and use distinct V-γ and V-δ gene segments. These fetal-derived γδ T-cells can be regarded as innate-like cells, which can respond rapidly without clonal expansion.

The adult thymus continues to produce γδ T-cells, which can be divided into distinct subsets by cell surface markers: as the CD27+ γδ population matures it downregulates expression of CD24, and upregulates CD44. The CD27+CD44+ population contains the NK1.1+ γδ subset (γδNKT cells) which can produce IL4 or IFNγ, and also includes cells that have a Th1-like bias of cytokine production (γδT1 cells), whereas the CD27-CD44+ population is enriched for Vγ2-gene usage and IL17-producing γδ cells (γδT17), and an additional adaptive γδ T-cell subset (γδTn) is also produced in the adult thymus (5–8). The early stages of γδ T-cell development in the thymus can be further subdivided by expression of cell surface CD25 and CD73, with the earliest γδTCR+ population being CD25+CD73-, and acquisition of cell-surface CD73 marking commitment to the γδ lineage (8–11). In contrast to αβT-helper cell differentiation in which naïve αβT-cells acquire effector function and phenotype following activation in the periphery, some γδ subsets are believed to be pre-committed for differentiation into their effector subset and functionally-programmed in the thymus (5–15).

Here we investigate the function of the Hedgehog (Hh) signaling pathway in γδ T-cell effector subset development in the thymus. The three mammalian Hedgehog family members (Sonic Hedgehog [Shh], Desert hedgehog [Dhh], and Indian hedgehog [Ihh]) signal by binding to their cell surface receptor Patched1 (Ptch1) (16). Ptch1 then releases its repression of the signal transducer Smoothened (Smo). At the end of the pathway are the Gli family of transcription factors (Gli1, Gli2 and Gli3) (17). Gli1 can only function as an activator of transcription and is itself a target gene of the signaling pathway. Gli2 and Gli3 are processed to act as transcriptional activators (Gli2A/Gli3A) when the signaling pathway is activated, or as transcriptional repressors (Gli2R/Gli3R) in the absence of Hh proteins.

The pathway has multiple positive and negative feed-back mechanisms (16, 17). *Ptch1* is itself a Hh target-gene, so that its upregulation can function to sequester Hh proteins and negatively regulate the pathway, the cell surface molecule Hedgehog interacting protein (Hhip) can also sequester Hh proteins, and the small GTP-binding protein Rab23 is a negative regulator of Hh signal transduction (18).

Shh signaling from thymic epithelial cells (TEC) to developing thymocytes promotes T-cell development at early stages of thymocyte development but negatively regulates αβ T-cell development at the pre-TCR and TCR-dependent transitions from DN to CD4+CD8+ double positive (DP) and DP to single positive (SP) cell (19–27). Smo is highly expressed in immature γδ T–cells (6) and conditional deletion of *Smo* from T-lineage cells, and constitutive Shh-deficiency in the fetal thymus reduced the overall production of γδ cells, consistent with the negative effect of Smo-deletion or Shh-deficiency on the DN2 population, but the influence of the Hh signaling pathway on γδ T-cell maturation and subset distribution in the thymus was not examined (19, 28). Hh signaling has also been shown to influence innate immune cell populations and to promote the proliferation and activation of murine liver iNKT cells (29–32).

Our study investigated the function of the Hh signaling pathway in γδ subset distribution in the thymus and in the homeostasis of γδ T-cell populations in the spleen. We show that Shh promotes γδ T-cell development in the thymus and is a determinant of γδ subset distribution, increasing the γδNKT population.

## Materials and Methods

### Mice

Gli2ΔN2-transgenic (tg) and Gli2ΔC2-tg were as described (23, 33). C57BL/6 mice were from Envigo. GBS-GFP-tg (34) were provided by J. Briscoe and Shh+/- (35) mice by P. Beachy. Mice were genotyped using methods and primers as described: GBS-GFP-tg (21); Gli2ΔN2-tg (23); Gli2ΔC2-tg (33); Shh+/- (19). Adult mice were between 4 and 6 weeks old. All mice were backcrossed onto a C57BL/6 background and bred and maintained at UCL. Mouse studies were approved by the British Home Office.

In some experiments, mice were treated by intraperitoneal (i.p.) injections with 40 μg/day of the Smo-inhibitor (Smo-inh) PF-04449913 (Pfizer) (36, 37) or vehicle control (DMSO) daily for 14 days.

### Flow Cytometry

Cells were stained as described (38), using antibodies from eBioscience (UK) and analyzed on a C6 Accuri flow cytometer (BD) or an LSR II (BD). Flow cytometry data were analyzed using FlowJo version 10.4.1 (Tree star). Live cells were gated according to FSC/SSC profiles.

### Cell Cultures

Fetal thymus organ cultures (FTOC) were carried out as described (27). In some experiments recombinant (r) Shh (R&D systems) or rHhip (Sigma) were added at 1 μg/ml.

### Quantitative (Q) RT PCR

Lymphocytes were sorted using a MoFlo (Cytomation, Fort Collins, CO). Cells collected fell within FSC/SSC live gate. RNA was extracted using Absolutely RNA miniprep kit (Agilent) or the PicoPure kit (Applied Biosystems). cDNA was synthesized using High Capacity cDNA reverse transcription kit (Applied Biosystems). cDNA samples were analyzed on the iCycler (Bio-Rad Laboratories, Hercules, CA) using SYBR Green Supermix (Bio-Rad) according to the manufacturer's guidelines. RNA levels obtained from each sample were measured relative to the level of the housekeeping gene *Hprt*, as described previously (31). Primers were purchased from QuantiTect (Qiagen).

### RNA Sequencing and Data Analysis

RNA sequencing was carried out as described (39). RNA was prepared from CD27+CD3+γδ+ cells sorted by FACS by UCL GOS ICH Flow cytometry facility (>95% purity) from the adult thymus and extracted using Arcturus PicoPure RNA Isolation kit (Applied Biosystems) and quantity and quality determined by Bioanalyser 2100 (Agilent). RNA was sequenced by UCL Genomics on the Illumina Next Seq 500. Genomic alignment was carried out by UCL Genomics using STAR v2.5b (via Illumina BaseSpace). The RNA sequencing dataset was processed and standardized using the Bioconductor package DESeq2, which was used to generate normalized estimates of transcript abundance, expressed as RPKM (reads per kilobase of transcript per million mapped reads). Data analysis was carried out as described (39, 40). Differentially expressed genes (DEG) were determined using moderated Ebayes *t*-statistic (*P* < 0.05) from the limma package in Bioconductor. Selection of transcription factors from DEG lists was carried out using PANTHER (41). Canonical Correspondence Analysis (CCA) was performed to compare the datasets to external publicly available datasets, as described in Ono et al. (42), using the CRAN package vegan. Heatmaps were generated using the CRAN package pheatmap and RColorBrewer: rows were centered; unit variance scaling was applied to rows; and rows were clustered using Pearson correlation distance and average linkage. Principal component analysis (PCA) was performed using normalized transcript expression values, using the built-in R function pca. The CRAN package factoextra was used to extract the list of contributing genes and then they were plotted using the package ggplot2. The RNA sequencing data are publicly available (GEO: GSE113468).

### Statistical Analysis

Unpaired two-tailed student's *t*-test using data from at least three independent experiments was used to test the significance of differences observed in WT and mutant mice, unless stated otherwise. ^*^p < 0.05; ^**^p < 0.01; ^***^p < 0.005.

## Results

### Hh Components Are Expressed by Thymic γδ T-Cells

**Figure 1:**
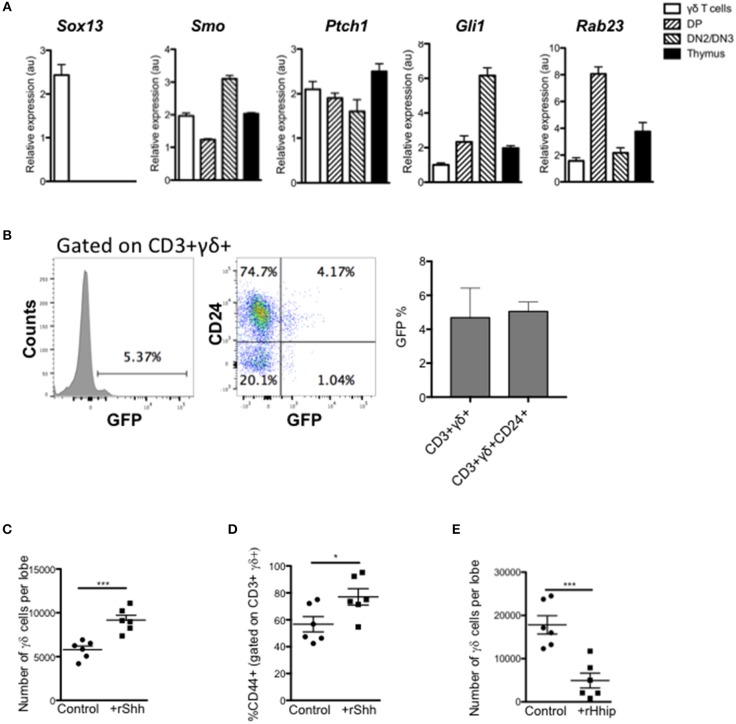
Hedgehog signaling promotes the γδ T-cell population in the thymus. **(A)** QRT-PCR analysis on CD3+ γδ+ (γδ cells), CD4+CD8+ (DP), CD4-CD8-CD25+ (DN2/DN3) populations (purified by FACS), and the whole unfractionated thymus (thymus) from 4 week-old WT mice. Expression of *Sox13, Smo, Ptch1, Gli1*, and *Rab23* are shown (relative to *Hprt*). Bar charts show mean ± SEM from three independent experiments (biological replicates). **(B)** Representative histogram shows GFP-fluorescence, gated on CD3+γδ+ cells from GBS-GFP-tg thymus, giving the percentage in the marker shown. Dot plot shows representative anti-CD24 staining against GFP-fluorescence from GBS-GFP-tg thymus, giving the percentage of cells in the quadrants shown. Bar charts show mean ± SEM percentage of GFP+ cells, gating on CD3+γδ+ and CD3+γδ+CD24+ cells from GBS-GFP-tg thymus (*n* = 3). **(C,D)** E14.5 WT FTOC were cultured for 5 days with rShh (1 μg/ml) or without treatment (control) and analyzed by flow cytometry. Each data point represents an individual thymus lobe. **(C)** Number of CD3+γδ+ cells recovered per thymus lobe for untreated (Control, *n* = 6) and rShh-treated (*n* = 6). **(D)** Percentage of CD44+ cells, gated on CD3+γδ+ cells recovered per thymus lobe for untreated (Control, *n* = 6) and rShh-treated (*n* = 6). **(E)** E16.5 WT FTOC were cultured for 5 days with rHhip (1 μg/ml) or without treatment (control) and analyzed by flow cytometry. Each data point represents an individual thymus lobe. Scatter plot shows the number of CD3+γδ+ cells recovered per thymus lobe for untreated (Control, *n* = 6) and rHhip-treated (*n* = 6). Each data point represents thymus from an individual embryo. ^*^p < 0.05; ^***^p < 0.005.

We first assessed if thymic γδ T-cells express components of the Hh signaling pathway and actively transduce Hh signals. We purified CD3+γδTCR+ cells by FACS from adult WT thymus and assessed gene expression by QRT-PCR, compared to FACS-purified CD4+CD8+ (DP) and CD25+CD4-CD8- (DN2/3) populations and unsorted thymus cell suspension (Figure 1A). As expected, the sorted γδ T-cells showed high expression of *Sox13*, which was not detected in other populations examined. We also detected expression of *Smo* [as expected (6)], and the Hh target genes and pathway components *Ptch1* and *Gli1* in γδ T-cells, and as previously reported in DP and DN2/3 cells (20, 23, 25, 28). The negative regulator *Rab23* was detected in all three subsets examined, with lower expression in the γδ T-cells and DN2/3 cells, and higher expression in the DP cells.

To confirm that thymic γδ T-cells transduce Hh signals *in vivo*, we made use of Gli binding site (GBS)-GFP-transgenic reporter mice (Hh reporter mice) (34). Gating on CD3+γδTCR+ thymocytes, we found that ~5% of thymic γδ T-cells expressed GFP, which reports Hh-mediated transcription (Figure 1B). The majority of GFP+ CD3+γδTCR+ thymocytes were CD24+, indicating that levels of Hh-mediated transcription were highest in immature γδ T-cells.

### Hh Signaling Promotes Fetal Thymic γδ T-Cell Production *in vitro*

Given that thymic γδ T-cells can transduce Hh signals, we tested the impact of treatment with recombinant (r) Shh and the Hh-neutralizing protein rHhip, on γδ T-cell development in FTOC. Treatment with rShh significantly increased the number of CD3+γδTCR+ cells recovered from WT FTOC, and significantly increased the proportion of the more mature CD44+ cells (Figures 1C,D). In contrast, treatment with rHhip significantly reduced the number of γδ T-cells recovered (Figure 1E). Thus, Hh signaling promoted γδ T-cell production. Interestingly, this is opposite to the effect of Hh on αβ T-cell development in FTOC, where Hh-neutralization promotes αβ T-cell maturation (22, 23, 26, 27), suggesting that Hh signaling may influence the γδ/αβ lineage choice.

### Shh Promotes γδ T-Cell Development and Increases the γδNKT Population in the Adult Thymus

**Figure 2:**
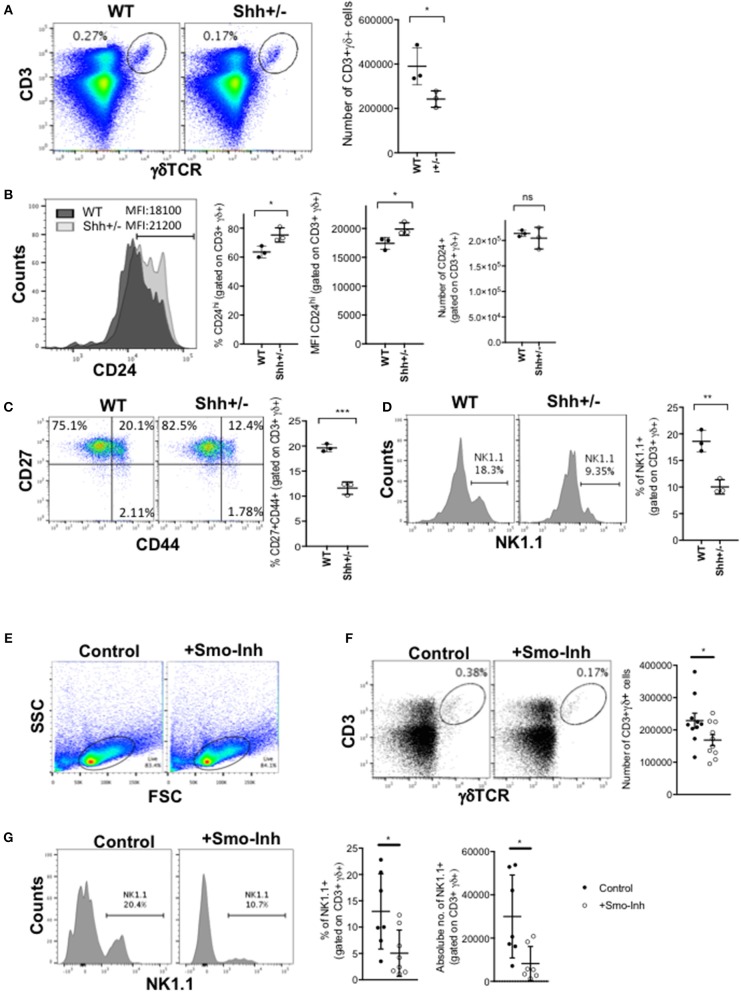
Shh and Smo promote γδ cell differentiation and influences γδ subset distribution in adult thymus. **(A–D)** Flow cytometry of adult Shh+/+ (WT, *n* = 3) and Shh+/- (*n* = 3) littermate thymus. **(A)** Dot plot shows anti-CD3 against anti-γδ staining from representative WT and Shh+/- littermate thymus, giving the percentage of cells in the region shown. Scatter plot shows the number of CD3+γδ+ cells recovered. **(B)** Representative histogram overlays show anti-CD24 staining, gated on CD3+γδ+ in WT (dark shade) and Shh+/- (light shade) adult thymus, giving the MFI. Left-hand scatter plot shows the percentage of CD24^hi^ cells (that fall within the marker shown on the histogram). Middle scatter plot shows MFI of anti-CD24 staining gated on CD3+γδ+ cells. Right-hand scatter plot shows the number of CD24+CD3+γδ+ cells in WT and Shh+/- littermate thymus. **(C)** Representative dot plot shows CD27 and CD44 expression, gated on CD3+γδ+ cells, giving the percentage of cells in the quadrants. The scatter plot shows the percentage of CD27+CD44+ cells, gating on CD3+γδ+ cells. **(D)** Representative histogram shows NK1.1 expression, gated on CD3+γδ+ cells, giving the percentage of cells in the marker. The scatter plot shows the percentage of NK1.1+ cells, gating on CD3+γδ+ cells. **(E–G)** Adult WT mice were injected ip with 40 μg/day of the Smo-inhibitor (Smo-Inh) PF-04449913 (Pfizer) (*n* = 10) or vehicle control (DMSO) (*n* = 10) daily for 14 days and thymus analyzed by flow cytometry. **(E)** Representative dot plot shows FSC vs. SSC to show the live gate used. **(F)** Representative dot plot shows CD3 and γδ expression in control and Smo-Inh-treated thymus. Scatter plot shows number of CD3+γδ+ cells recovered per thymus from control and Smo-Inh-treated mice. **(G)** Representative histogram shows NK1.1 expression, gated on CD3+γδ+ cells from control and Smo-Inh-treated thymus. Scatter plots show the percentage (left) and number (right) of NK1.1+ cells, gated on CD3+γδ+ cells, per thymus from control and Smo-Inh-treated mice. In all scatter plots in this figure, each point represents an individual mouse and bars show mean ± SEM. ^*^p < 0.05; ^**^p < 0.01; ^***^p < 0.005.

We next investigated if Hh signaling also increased γδ T-cell populations in adult thymus. As Shh-deficiency is embryonic lethal, we examined the thymus of adult Shh+/- mice. The adult Shh+/- thymus contained significantly fewer γδ T-cells than WT (Figure 2A). Cell surface CD24 expression was higher on the CD3+γδTCR+ population, indicating a less mature phenotype (Figure 2B). Interestingly, the number of CD24+CD3+γδTCR+ cells (cells that stained highly and fell within the positive marker shown in the histogram) recovered from the Shh+/- thymus was not significantly lower than in the WT, showing that in the Shh+/- thymus the γδ population is enriched for immature cells and the more mature CD24low/negative γδ T-cell population is reduced (Figure 2B). Consistent with this, within the Shh+/- CD3+γδTCR+ population, there was a significant decrease in the proportion of CD27+CD44+ cells compared to WT (Figure 2C), and the Shh+/- CD3+γδTCR+ population contained significantly fewer γδNKT cells than WT (Figure 2D).

### Pharmacological Smo-Inhibition Reduces Adult Thymic γδ T-Cell Populations *in vivo*

We then tested if in adult mice, inhibition of the Hh pathway *in vivo* by treatment with a pharmacological Smo-inhibitor influenced γδ T-cell populations in the WT thymus. After 14 days treatment *in vivo* by i.p. injection, the CD3+γδTCR+ population in the thymus was significantly decreased in the Smo-inhibitor-treated mice, compared to vehicle (DMSO)-treated controls (Figures 2E,F). Treatment with the Smo-inhibitor also caused a significant reduction in the proportion and number of γδNKT cells in the thymus (Figure 2G), consistent with the Shh+/- adult thymus (Figure 2D).

### Hh-Mediated Transcription in T-Lineage Cells Promotes γδ T-Cell Maturation

**Figure 3:**
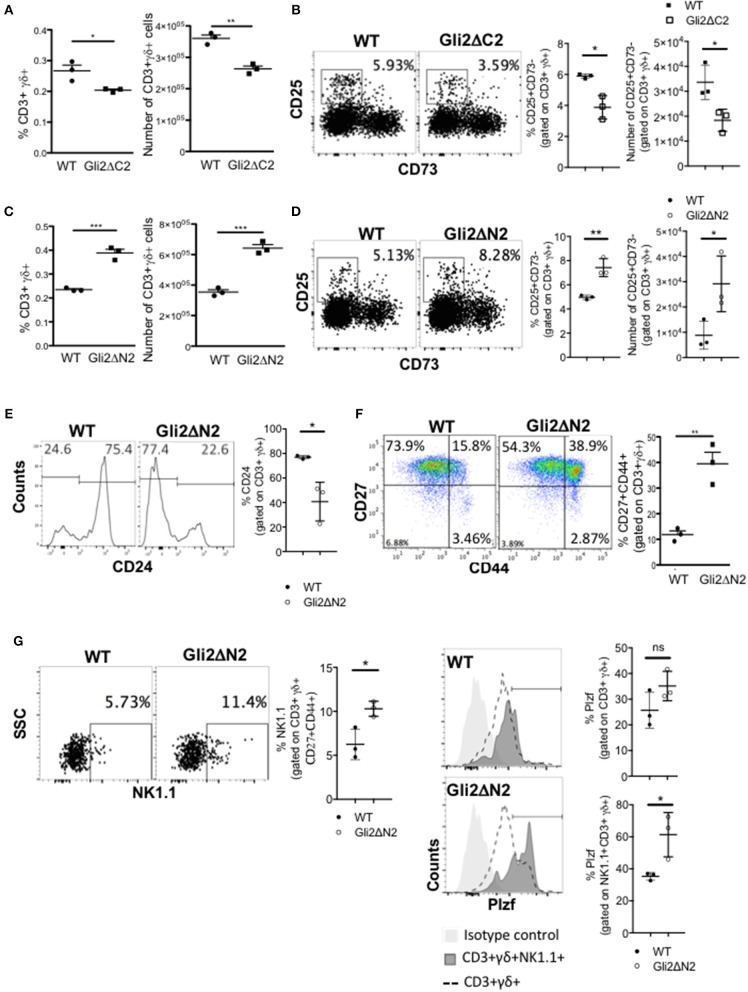
Hh-mediated transcription promotes γδ T-cell development and influences γδ subset distribution in adult thymus. **(A)** The proportion and number of CD3+γδ+ cells recovered from WT (*n* = 3) and Gli2ΔC2-tg (*n* = 3) littermate adult thymus. **(B)** Representative dot plots show CD25 and CD73 expression, gated on CD3+γδ+ cells from WT and Gli2ΔC2-tg, giving the percentage of cells in the CD25+CD73- region shown. Scatter plots show the percentage of CD25+CD73- cells, gated on CD3+γδ+ (left) and number of CD25+CD73-CD3+γδ+ cells recovered (right) from WT (*n* = 3) and Gli2ΔC2-tg (*n* = 3). **(C–G)** Flow cytometry analysis of WT (*n* = 3) and Gli2ΔN2-tg (*n* = 3) littermate adult thymus. **(C)** The proportion and number of CD3+γδ+ cells recovered. **(D)** Representative dot plots show CD25 and CD73 expression, gated on CD3+γδ+ cells from WT and Gli2ΔN2-tg, giving the percentage of cells in the CD25+CD73- region shown. Scatter plots show the percentage of CD25+CD73- cells, gated on CD3+γδ+ (left) and number of CD25+CD73-CD3+γδ+ cells recovered (right). **(E)** Representative histograms show anti-CD24 staining, gated on CD3+γδ+. Scatter plots show the percentage of CD24+ cells, gated on CD3+γδ+. **(F)** Representative dot plot shows anti-CD27 and anti-CD44 staining, gated on CD3+γδ+, giving the percentage of cells in each quadrant. Scatter plot shows the percentage of CD27+CD44+ cells, gated on CD3+γδ+. **(G)** Representative dot plot shows anti-NK1.1 staining against SSC, gated on CD3+γδ+CD44+CD27+, giving the percentage of NK1.1+ cells in the region shown. Middle scatter plot shows the percentage of NK1.1+ cells, gated on CD3+γδ+CD44+CD27+. Representative histogram overlays show intracellular anti-PLZF staining, gated on CD3+γδ+ (dotted lines) and on CD3+γδ+NK1.1+ (dark shading) and isotype control staining (light shade) on WT (upper) and Gli2ΔN2-tg (lower) thymus. Scatter plots how the percentage of PLZF+ cells (cells within the marker in the histogram), gated on CD3+γδ+ (upper plot) and on CD3+γδ+NK1.1+ (lower plot). In all scatter plots in this figure, each point represents an individual mouse and bars show mean ± SEM. ^*^p < 0.05; ^**^p < 0.01; ^***^p < 0.005.

As rShh-treatment increased γδ T-cell populations in FTOC, and Smo-inhibition and Shh-mutation reduced γδ T-cells in adult thymus *in vivo*, we next tested if these changes were the direct result of Hh pathway activation in developing T-lineage cells or were due to indirect effects by other cell types in the thymus. To do so, we used transgenic mice which express modified forms of Gli2 that can function as transcriptional activator only (Gli2ΔN2-tg) or transcriptional repressor only (Gli2ΔC2-tg) in T-lineage cells from the DN2 stage onwards (23, 33). Inhibition of normal physiological levels of Hh-mediated transcription in the adult Gli2ΔC2-tg thymus significantly reduced the proportion and number of CD3+γδTCR+ cells in the thymus compared to WT (Figure 3A), and the proportion and number of the earliest CD3+γδ+CD25+CD73- stage was also significantly reduced (Figure 3B). In contrast, increasing Hh-mediated transcription in thymocytes to above WT levels in the adult Gli2ΔN2-tg led to a significant increase in the proportion and number of CD3+γδTCR+ cells, and the proportion and number of the earliest CD3+γδ+CD25+CD73- population was also significantly increased (Figures 3C,D). Active Hh-mediated transcription also influenced later stages of γδ T-cell maturation and differentiation. We observed a significant decrease compared to WT in the proportion of immature CD24^high^ cells, gating on CD3+γδTCR+ (Figure 3E), whereas the proportion of CD27+CD44+ cells was significantly increased (Figure 3F). The CD3+γδ+NK1.1+CD27+CD44+ (γδNKT) population was significantly increased in the Gli2ΔN2-tg thymus compared to WT (Figure 3G), and expression of the transcription factor PLZF, which is required for γδNKT cell development and function (43), was significantly higher in the CD3+γδ+NK1.1+ population in the Gli2ΔN2-tg thymus (Figure 3G). Thus, increased Hh-mediated transcription in thymocytes to above WT levels led to an overall increase in the number of γδ T-cells in the adult thymus (Figure 3C), and also changed the subset distribution within the CD3+γδ+ population, with an increase in the earliest CD25+CD73- population, consistent with the overall increase in γδ T-cells, and also an increase in the PLZF+ γδNKT population.

### Hh Signaling Influences the Maturation Status and Transcriptional Signature of Thymic γδ Populations

**Figure 4:**
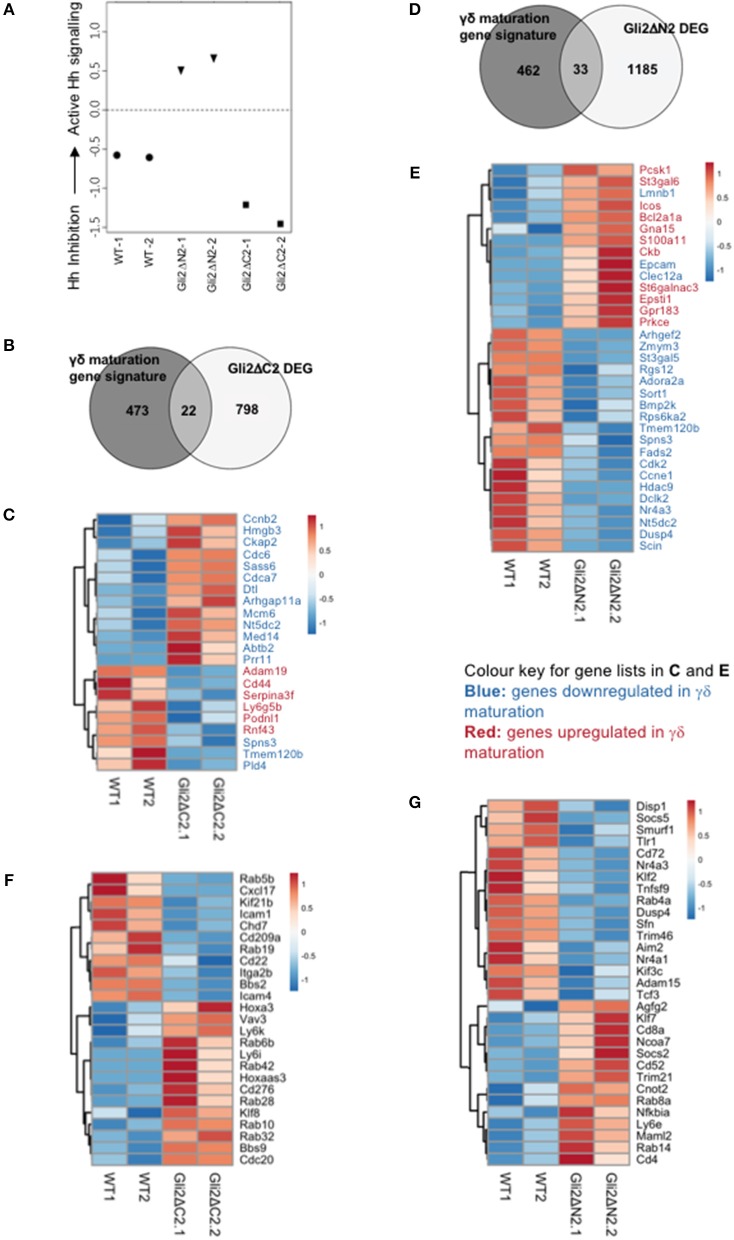
Influence of Hh signaling on the transcriptional pattern of thymic CD27+CD3+γδ+ cells. RNA sequencing was carried out on CD27+CD3+γδ+ thymocytes (purified by FACS) from WT, Gli2ΔN2-tg, and Gli2ΔC2-tg. Two datasets from independent sorts (biological replicates) were carried out for each genotype. **(A)** Canonical correspondence analysis (CCA) was used to generate a scale of Hh-inhibition to active Hh signaling and our datasets were plotted against this scale, where the negative half of the y-axis represents Hh-inhibition and the positive half increasing score of active Hh signaling. **(B)** The Venn diagram illustrates the intersection between the DEG list of Gli2ΔC2-vs.-WT with the published list of γδ maturation signature genes to identify genes that are regulated by physiological levels of Hh signaling and also contribute to γδ T-cell maturation. **(C)** The Pearson correlation clustering heatmap shows expression in WT and Gli2ΔC2 of the 22 DEG between Gli2ΔC2-vs.-WT from the intersection in **(B)**, where red represents higher expression and blue lower expression on a linear correlation scale. A value of 1 indicates a positive association, while a value of −1 indicates a negative association, and a value of 0 indicates no association. Gene names given in blue are genes that are downregulated during γδ maturation, whereas gene names given in red are genes which are upregulated during γδ maturation. **(D)** The Venn diagram illustrates the intersection between the DEG list of Gli2ΔN2-vs.-WT with the published list of γδ maturation signature genes to identify genes that are influenced by increasing levels of Hh signaling and also contribute to γδ T-cell maturation. **(E)** The Pearson correlation clustering heatmap shows expression in WT and Gli2ΔN2 of the 33 DEG between Gli2ΔN2- vs.-WT from the intersection in **(D)**, where red represents higher expression and blue lower expression on a linear correlation scale. A value of 1 indicates a positive association, while a value of −1 indicates a negative association, and a value of 0 indicates no association. Gene names given in blue are genes that are downregulated during γδ maturation, whereas gene names given in red and genes which are upregulated during γδ maturation. **(F)** The Pearson correlation clustering heatmap shows expression of selected DEG (*P* < 0.05 by Ebayes) from WT and Gli2ΔC2 datasets where red represents higher expression and blue represents lower expression on a linear correlation scale. A value of 1 indicates a positive association, while a value of −1 indicates a negative association, and a value of 0 indicates no association. **(G)** The Pearson correlation clustering heatmap from WT and Gli2ΔN2 datasets shows expression of selected DEG (*P* < 0.05 by Ebayes) where red represents higher expression and blue represents lower expression on a linear correlation scale. A value of 1 indicates a positive association, while a value of −1 indicates a negative association, and a value of 0 indicates no association.

**Figure 5:**
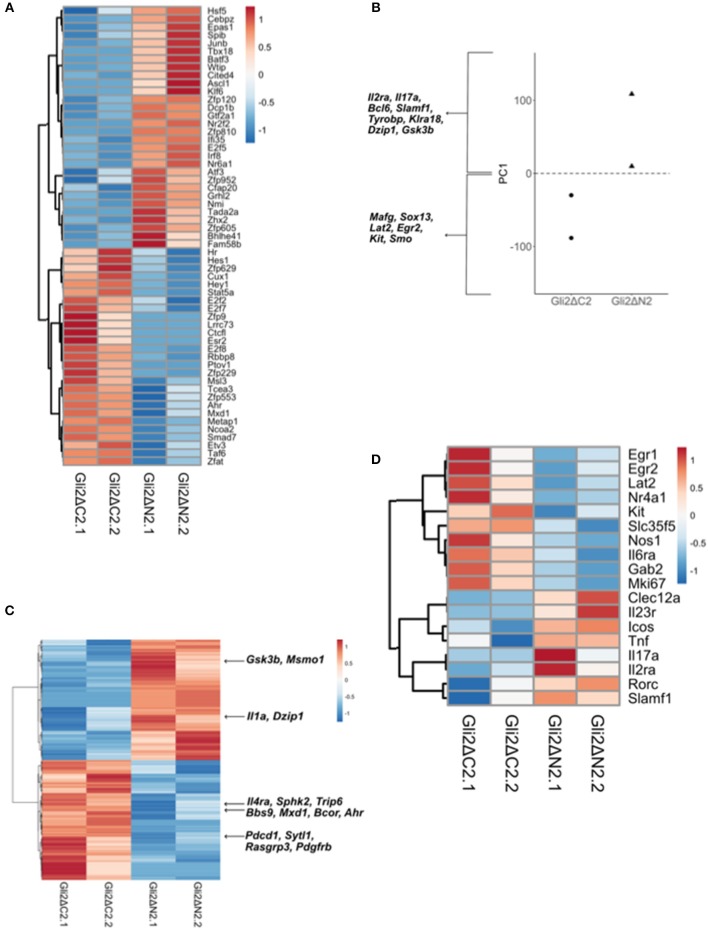
Comparison of Gli2ΔN2 and Gli2ΔC2 datasets to investigate the influence of Hh signaling on the transcriptional pattern of thymic CD27+CD3+γδ+ cells. Further analysis of the RNA sequencing datasets (as in Figure 4): two datasets from independent FACS sorts (biological replicates) of CD27+CD3+γδ+ thymocytes from Gli2ΔN2-tg and Gli2ΔC2-tg thymus. **(A)** The Pearson correlation clustering heatmap shows expression in Gli2ΔN2 and Gli2ΔC2 of 57 DEG that encode transcription factors (identified by PANTHER), where red represents higher expression and blue lower expression on a linear correlation scale. A value of 1 indicates a positive association, while a value of −1 indicates a negative association, and a value of 0 indicates no association. **(B)** Principle component axis 1 (PC1) for Gli2ΔN2 and Gli2ΔC2 DEG, showing some genes that contributed to the negative (Gli2ΔC2) and positive (Gli2ΔN2) PC1 axis. **(C)** Pearson correlation clustering heatmap of DEGs identified by intersection analysis between the DEG (*P* < 0.05 by Ebayes) and genes that highly contributed to the negative and positive PC1 axis (genes that scored >0.80 and <-0.80), illustrating some genes that contribute to γδ T-cell maturation and Hh signaling. Red represents higher expression and blue represents lower expression on a linear correlation scale. A value of 1 indicates a positive association, while a value of −1 indicates a negative association, and a value of 0 indicates no association. **(D)** The Pearson correlation clustering heatmap from Gli2ΔC2 and Gli2ΔN2 datasets shows expression of selected DEG (*P* < 0.05 by Ebayes) where red represents higher expression and blue lower expression on a linear correlation scale. A value of 1 indicates a positive association, while a value of −1 indicates a negative association, and a value of 0 indicates no association.

To investigate the impact of Hh pathway activation on the transcriptional signature and differentiation of thymic γδ populations, we carried out RNA sequencing on CD3+γδTCR+CD27+ cells purified by FACS from Gli2ΔN2-tg, Gli2ΔC2-tg and WT thymus (Figures 4, 5).

In order to confirm that both transgenes were active in developing γδ T-cells and were able to inhibit or increase the levels of Hh-mediated transcription, we first used canonical correspondence analysis (CCA) to compare our datasets to publicly available transcriptome datasets: we generated a scale of inhibited-to-active Hh signaling, using transcriptome datasets from resting CD4 T-cells from Gli2ΔN2-tg and Gli2ΔC2-tg [(44) GEO: GSE33156]. As expected, when we plotted the six samples against this scale, the samples segregated by genotype, with the Gli2ΔN2-tg γδ T-cells scoring highly on the Hh-activated scale, the Gli2ΔC2-tg γδ T-cells scoring highly in the inhibition-of-Hh signaling axis, and WT samples scoring intermediate (Figure 4A).

The datasets contained many differentially expressed genes (DEG) between genotypes, with levels of Hh-mediated transcription influencing expression of genes associated with differentiation, signaling, and lymphocyte function. Therefore, in order first to determine the transcriptional influence of normal levels of Hh signaling on thymic CD3+γδTCR+CD27+ cells, we used EBayes statistics to identify 820 DEG genes between Gli2ΔC2 and WT datasets (Supplementary Table 1). Then, to investigate if these Hh-regulated genes are important in γδ T-cell maturation, we intersected the 820 DEG with a list of γδ maturation signature genes (495 genes) that increase or decrease upon maturation of γδ T-cells, as defined by a published transcriptome analysis of immature γδ T-cells (CD24^hi^) and mature γδ T-cells (CD24^lo^) from adult thymus from the Immunological Genome Project Consortium (6). The intersection revealed 22 common elements (Figure 4B), 13 of which were more highly expressed in the Gli2ΔC2 datasets than WT and therefore are normally down-regulated by Hh signaling, and these were all genes that have been shown to be downregulated as γδ T-cells mature. Nine DEG, however, showed lower expression in the Gli2ΔC2 datasets, and so are normally upregulated by Hh signaling. Six of these, including *Cd44*, are upregulated as γδ T-cells mature (Figure 4C). Thus, the transcriptional signature induced by physiological levels of Hh signaling in the CD27+CD3+γδ+ thymocyte population promoted γδ T-cell maturation, but inhibition of Hh-mediated transcription also affected the expression of many genes that have not been associated with γδ T-cell maturation.

Next, we investigated how increasing levels of Hh-mediated transcription to above normal WT levels influenced the pattern of gene expression, by comparison of Gli2ΔN2 and WT datasets. Ebayes statistics identified 1218 DEG (Supplementary Table 2) and intersection of these with the 495 γδ maturation signature genes highlighted 33 genes which are regulated by increasing the level of Hh signaling and are also important in γδ maturation (Figure 4D). Nineteen of these DEG were downregulated in the Gli2ΔN2 datasets and importantly these are also all genes that are known to be downregulated as γδ thymocytes mature (Figure 4E). These down-regulated genes included *Nr4a3*, which is a transcriptional target of TCR-signaling (45), so its down-regulation is consistent with the reduced TCR-signal strength observed in Gli2ΔN2-tg thymocytes (23, 25). In contrast, of the 14 intersection genes that had higher expression in the Gli2ΔN2 datasets, 11 were genes that are also upregulated during γδ T-cell maturation, including *ICOS*, which is highly expressed in γδNKT lineage cells in the thymus (8) and can signal to reduce development of the γδ17 population (46). Increased expression of *Clec12a* (*Cd371*), was consistent with the increase in CD25+CD73-CD3+γδ+ cells in the Gli2ΔN2-tg thymus (Figure 3D), as it is highly expressed on the most immature γδ populations, and its expression remains high in precursors of the γδTn population (8). Taken together, the DEG between Gli2ΔC2-vs.-WT datasets, and between Gli2ΔN2- vs.-WT datasets both indicate that during γδ T-cells maturation in the thymus Hh pathway activation signals to promote maturation, both by downregulation of genes associated with immature γδ T-cell populations and upregulation of genes required for maturation. This is supported by our FTOC and *ex vivo* analysis of thymic γδ T-cells populations. Increasing the Hh signal (by rShh treatment; and Gli2ΔN2 transgene expression) increased maturation of γδ T-cells in the thymus. In contrast, reduction in Hh signaling to below normal physiological levels (by Shh-mutation; Gli2ΔC2 transgene expression; rHhip treatment *in vitro*; and Smo-inhibitor treatment *in vivo*) reduced the γδ T-cell populations in the thymus.

Further comparison between the Gli2ΔC2-tg and WT datasets revealed many genes important in signal transduction or immune function that were regulated by inhibition of physiological levels of Hh-mediated transcription in CD27+CD3+γδ+ cells (Figure 4F). Amongst down-regulated DEG were the chromatin remodeling gene *Chd7*; the microtubule-associated motor protein *Kif21b* and the BBSome complex member *Bbs2*, both of which are involved in cilia transport and morphogen signaling; the chemokine *Cxcl17;* members of the Rab GTPase family (*Rab5b, Rab19*), which function as regulators of intracellular vesicle transport; and several adhesion molecules (*Icam*1, *Cd22, Cd209a, Itga2b*, and *Icam4*). DEG upregulated in Gli2ΔC2-tg compared to WT datasets also included several members of the Rab GTPase family (*Rab10, Rab6b, Rab42, Rab28, Rab32*); the homeobox gene *Hoxa3*; BBSome complex member *Bbs9*; the B7 family member *Cd276;* and the transcription factor *Klf8*. The guanine nucleotide exchange factor *Vav3* was also upregulated when normal levels of Hh-mediated transcription were reduced, consistent with previous findings that Hh pathway activation can reduce TCR signal strength in αβ T-cells, and with its higher expression in immature γδ T-cells (6, 21, 23, 25, 26, 33, 47, 48).

Increased Hh-mediated transcription to above normal levels, in the Gli2ΔN2-tg CD27+CD3+γδ+ thymocytes also influenced levels of expression of several Rab family members (down-regulation of *Rab4a*, upregulation of *Rab14*, and *Rab8a*); and influenced expression of many genes associated with immune function and/or TCR signaling (including downregulation compared to WT of *Tnfsf9, Cd72, Dusp4, Trim46, Nr4a3, Nr4a1, Tcf3, Socs5*; upregulation *of Nfkbia, Cd8a, Cd4, Socs2, Cd52, Trim21*); and influenced genes associated with Hedgehog (*Kif3c, Disp1*) and Notch (*Adam15, Maml2*) pathways (Figure 4G). Expression of the transcription factor *Klf2* was significantly lower in the Gli2ΔN2-tg datasets than WT (Figure 4G). This is of interest given that Klf2 is required for γδ T-cell thymic egress and its deficiency results in an increase in the incidence of γδNKT cells, many of which expressed CD4 (49).

To investigate further the influence Hh-mediated transcription on thymic γδ T-cell development we then filtered DEG between Gli2ΔC2 and Gli2ΔN2 datasets for genes that encode transcription factors, in order to identify potential developmental regulators down-stream of Hh-mediated transcription (Figure 5A). Expression of the Notch target genes *Hes1* and *Hey1* were higher in the Gli2ΔC2 datasets, suggesting that inhibition of Hh pathway activation led to increased Notch signal transduction, of interest given that the Notch-Hes1 pathway is required for development of γδ17 cells (50). Interestingly, *Ahr* was also higher in the Gli2ΔC2 datasets, and high levels of *Ahr* expression are associated with IFNγ-producing γδ intraepithelial lymphocytes (51, 52) and the IFNγ-producing non-NKT γδ population (γδT1) in the thymus (8).

Next, to compare Gli2ΔC2 and Gli2ΔN2 datasets in an unbiased manner, we carried out Principal component analysis (PCA) on normalized expression values between the datasets. PCA separated datasets by genotype on Principal component (PC)1 (Figure 5B), which accounted for 43.76% of variance. Genes that contributed strongly to the positive axis (with higher expression in Gli2ΔN2 datasets) included: *Bcl6*; Hh-associated genes (including *Dzip1* and *Gsk3b*); and NK-associated genes (including *Klra18* and *Tyrobp*). Genes that contributed strongly to the negative axis (higher expression in Gli2ΔC2 datasets) included *Sox13* and the Hh signal transduction molecule *Smo*, which are both more highly expressed in immature thymic γδ populations (6, 8). To highlight DEG that are important for the differences between genotypes, we intersected the genes that contributed most to PC1 with the DEG (*p* < 0.05) between Gli2ΔC2 and Gli2ΔN2 datasets (Figure 5C). DEG of interest included *Il1a*, which was more highly expressed when Hh-mediated transcription was increased, whereas genes that were increased when Hh-mediated transcription was inhibited included *Il4ra*, the Bcl6-co-repressor *Bcor*, the Myc-pathway gene *Mxd1*.

Finally, we selected DEG whose level of expression is known to correspond to distinct stages of γδ T-cell development or γδ T-cell subsets or to be induced by γδTCR signaling in the thymus (8, 53). Expression of *Egr*1, *Egr2, Nr4a1, Mki67*, which have been shown to be upregulated by TCR signaling, were higher in Gli2ΔC2 cells, as were the signaling molecules *Lat2* and *Gab2* (Figure 5D). Constitutive Hh-mediated transcription led to upregulation of genes that are highly expressed by the thymic γδNKT population (*Il23r, Icos, Tnf*, *Il17a*), supporting the notion that Hh pathway activation promotes the γδNKT population in the thymus (Figure 5D). We also observed increased expression of *Rorc, Slamf1, and Clec12a* (*Cd371*), which are more highly expressed in the adult thymic precursors of the TCR-naive adaptive γδ T-cells (γδTn) (8).

### Influence of Hh Signaling on Spleen γδ Subsets

**Figure 6:**
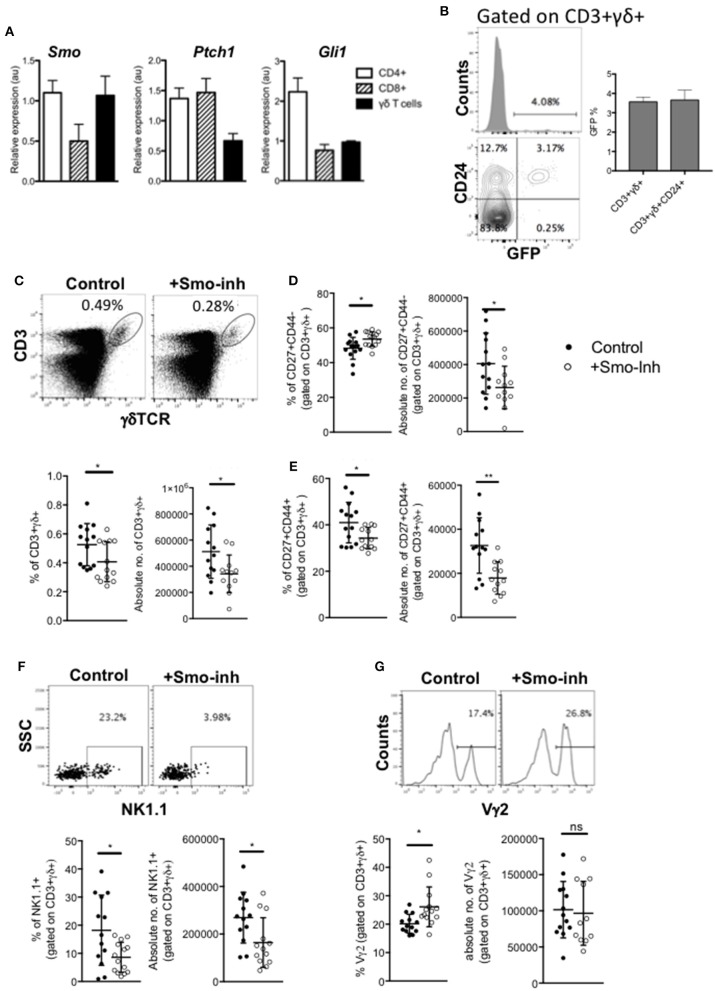
Hh signaling is active in spleen γδ cells and influences spleen γδ subset distribution and homeostasis. **(A)** QRT-PCR analysis on CD3+γδ+, CD3+CD4+CD8-, and CD3+CD4-CD8+ populations (purified by FACS) from spleen from 4 week-old WT mice. Expression of *Smo, Ptch1*, and *Gli1* are shown (relative to *Hprt*). Bar charts show mean ± SEM from three independent experiments (biological replicates). **(B)** Representative histogram shows GFP-fluorescence, gated on CD3+γδ+ cells from GBS-GFP-tg spleen, giving the percentage of GFP+ cells in the marker shown. Representative contour plot shows anti-CD24 staining against GFP-fluorescence from GBS-GFP-tg spleen, giving the percentage of cells in the quadrants. Bar chart shows the mean ± SEM percentage of GFP+ cells, gated on CD3+γδ+ cells, and gated on CD3+γδ+CD24+ cells from GBS-GFP-tg (*n* = 3) spleen. **(C–G)** Adult WT mice were injected ip with 40μg/day of the Smo-inhibitor (Smo-inh) PF-04449913 (Pfizer) (*n* = 13) or vehicle control (DMSO) (*n* = 13) daily for 14 days and spleen analyzed by flow cytometry. **(C)** Representative dot plot shows anti-CD3 and anti-γδ staining on spleen cells from control and Smo-inhibitor treated mice, giving the percentage of CD3+γδ+ cells in the region shown. Scatter plots show the percentage and number of CD3+γδ+ cells in the spleen. **(D)** Scatter plots show the percentage and number of CD27+CD44- cells, gated on CD3+γδ+ from control and Smo-inhibitor treated mice. **(E)** Scatter plots show the percentage and number of CD27+CD44+ cells, gated on CD3+γδ+ in the spleen from control and Smo-inhibitor treated mice. **(F)** Representative dot plot shows anti-NK1.1 staining vs. SSC, gated on CD3+γδ+ spleen cells from control and Smo-inhibitor treated mice, giving the percentage of cells in the region shown. Scatter plots show the percentage and number of NK1.1+ cells, gated on CD3+γδ+ in the spleen from control and Smo-inhibitor treated mice. **(G)** Representative histogram shows anti-Vγ2 staining, gated on CD3+γδ+ cells in the spleen from control and Smo-inhibitor treated mice. Scatter plots show the percentage and number of Vγ2+ cells, gated on CD3+γδ+ cells in the spleen from control and Smo-inhibitor treated mice. In all scatter plots in this figure, each point represents an individual mouse and bars show mean ± SEM. ^*^p < 0.05; ^**^p < 0.01.

As mutation of Hh pathway components caused profound changes in the transcriptional signature of CD27+CD3+γδTCR+ cells in the thymus, and Hh signaling increased the thymic γδNKT population, which migrate to the spleen, we investigated γδ T-cell subsets in the spleen. First, to test if they respond to Hh signals, we compared expression of *Ptch1, Smo*, and *Gli1* by QRT-PCR on RNA prepared from WT splenic FACS-purified CD3+γδ+ cells to their expression in FACS-purified CD4+ and CD8+ αβT-cells, which are known to express Hh pathway components and transduce Hh signals (23, 47, 54). *Smo* was expressed at similar levels in γδ T-cells as in the CD4+ αβ T-cell population, whereas expression of the target genes *Ptch1* and *Gli1* were lower than in the αβ T-cell populations (Figure 6A). Analysis of the GBS-GFP transgenic spleen indicated that ~4% of γδ T-cells expressed GFP (reporting Gli-mediated transcription). The majority of GFP+ γδ T-cells belonged to the less mature CD24+ subset, suggesting that levels of Hh-mediated transcription were highest in immature γδ T-cells in the spleen (Figure 6B).

Therefore, to test if splenic γδ T-cell homeostasis is still sensitive to modulation of Hh pathway activation, we investigated the impact of systemic Smo-inhibition on splenic γδ+ populations *in vivo*, after 14 days treatment with Smo-inhibitor or DMSO-control. Smo-inhibition led to a significant reduction in the proportion and number of CD3+γδ+ cells in the spleen (Figure 6C). When gating on the CD3+γδ+ population, we observed a significant increase in the proportion of CD27+CD44- cells in the Smo-inhibitor treated group compared to control, although the number of CD27+CD44-CD3+γδ+ cells was in fact lower than in the control, as a result of the overall decrease in CD3+γδ+ cells (Figure 6D). There was a significant decrease in the proportion of CD27+CD44+ cells and a significant decrease in their number (Figure 6E). The proportion and number of CD3+γδ+NK1.1+ cells were also significantly reduced in spleen (Figure 6F), whereas the proportion of Vγ2+ cells (gated on CD3+γδ+) was significantly increased in the spleen upon Smo-inhibition while the number of CD3+γδ+Vγ2+ cells was not significantly different from control (Figure 6G). Thus, the Smo-inhibitor treatment changed the γδ subset distribution in the spleen, and particularly reduced the γδNKT population, but did not appear to affect the Vγ2+ population.

**Figure 7:**
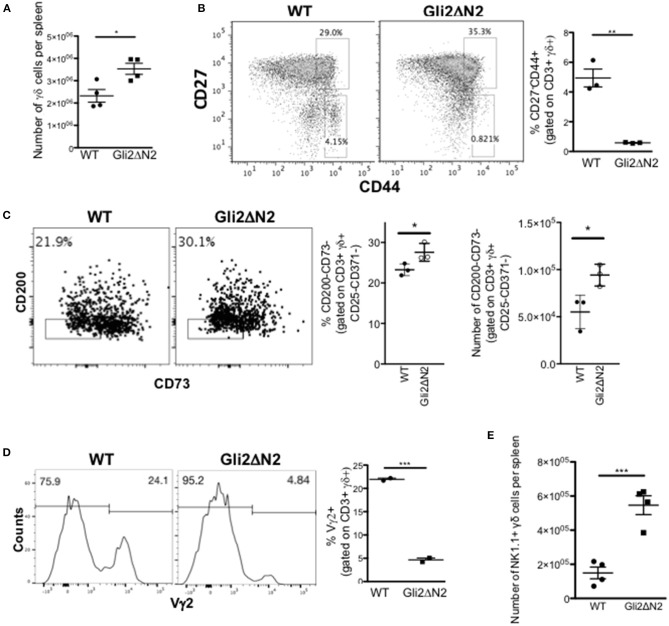
Hh-mediated transcription in γδ T-cells influences γδ T-cell populations in adult spleen. **(A–D)** Gli2ΔN2-transgenic and WT littermate spleens were analyzed by flow cytometry. In scatter plots each point represents data from a different mouse; bars show mean ± SEM. **(A)** The number of CD3+γδ+ cells in the spleen. **(B)** Representative dot plot shows anti-CD27 and anti-CD44 staining gated on CD3+γδ+ cells from WT and Gli2ΔN2-tg spleen, giving the percentage of cells in the regions shown. Scatter plot shows the percentage of CD27-CD44+ cells, gated on CD3+γδ+ cells in the spleen. **(C)** Representative dot plot shows anti-CD200 and anti-CD73 staining, gated on CD3+γδ+CD25-CD371- cells from WT and Gli2ΔN2-tg spleen, giving the percentage of cells in the region shown. Scatter plots show the percentage of CD200-CD73- cells, gated on CD3+γδ+CD25-CD371- cells, and the number of CD200-CD73-CD3+γδ+CD25-CD371- cells in WT and Gli2ΔN2-tg spleen. **(D)** Representative histograms show anti-Vγ2 staining, gated on CD3+γδ+ cells in the spleen, giving the percentage of cells in the marker shown. Scatter plots shows the percentage of Vγ2+ cells, gated on CD3+γδ+ cells in the spleen. **(E)** Scatter plot shows the number of NK1.1+ cells, gated on CD3+γδ+ cells in the WT and Gli2ΔN2-tg spleen. ^*^p < 0.05; ^**^p < 0.01; ^***^p < 0.005.

In contrast, the γδ population and CD27/CD44 subset distribution in the Gli2ΔN2-tg spleen mirrored that of the Gli2ΔN2-tg thymus and was consistent with the thymus transcriptome data, with a significant increase in the number of γδ T-cells in the spleen, but a significant decrease in the proportion of CD44+CD27- cells (Figures 7A,B). As transcription of *Rorc, Slamf1, and Clec12a* were increased in the Gli2ΔN2 CD27+CD3+γδ+ population (Figure 5D), we examined the TCR-naive γδTn, which have been described to be CD3+γδ+CD25-CD371-CD200-CD73- cells (8). The proportion and number of this population were significantly increased in the Gli2ΔN2 spleen compared to WT, suggesting that increased Hh pathway activation promotes their development or proliferation (Figure 7C). Gating on CD3+γδ+ cells, the proportion of Vγ2+ cells was decreased by more than 4-fold (Figure 7D), consistent with the significant reduction in the CD27-CD44+ population (Figure 7B). As in the thymus, the γδNKT population was significantly increased by more than 2-fold in the Gli2ΔN2 spleen compared to WT (Figure 7E).

## Discussion

Here we show that Shh signaling in the thymus is a determinant of γδ T-cell maturation and subset distribution. Increased Hh signaling *in vivo* by constitutive expression of a transgenic activator-only form of Gli2 in T-lineage cells, and *in vitro* by rShh treatment of FTOC promoted maturation of γδ T-cells in the thymus. In contrast, reduction in Hh pathway activation *in vivo*, by conditional inhibition of normal Hh-mediated transcription in T-lineage cells, by mutation of Shh, and by systemic pharmacological Smo-inhibition, reduced γδ T-cell populations in thymus and spleen. Hh pathway activation favored the γδNKT population in both thymus and spleen, but also influenced multiple stages of development and subsets, including the earliest immature CD25+CD73-CD3+γδ+ cells, and the subset distribution between CD27-CD44+Vγ2+, γδNKT, and γδTn populations.

Our experiments showed that Shh is required for adult thymic γδ T-cell maturation, as its mutation (in Shh heterozygote) not only led to a reduction in γδ T-cell numbers, but to an enrichment of the CD24^hi^ immature subset, and reduction in expression of the maturation marker CD44, as well as a reduced thymic γδNKT population. These effects were the result of direct signaling to developing thymocytes, as conditional inhibition of normal Hh-mediated transcription (in Gli2ΔC2 thymus) also reduced γδ T-cell numbers, and reduced the number and proportion of the earliest CD25+CD73- subset, indicating that Hh pathway activation promotes the earliest stages of γδ T-cell development. This was supported by the increase in this CD25+CD73- population when Hh-mediated transcription was increased to above normal levels in the Gli2ΔN2-tg thymus.

We used RNA sequencing to investigate the differentiation status of the thymic CD27+CD3+γδ+ population. This analysis confirmed that physiological levels of Hh signaling promote γδ T-cell development in the thymus, as inhibition or increase in normal levels of Hh-mediated transcription regulated expression of many genes that are involved in γδ T-cell maturation.

In αβ T-cells, Hh pathway activation has been shown to reduce TCR signal strength (47), and strong TCR signaling has been proposed to favor γδ T-cell fate over αβ T-cell fate in DN cells (2). However, although our RNA sequencing datasets also indicated that Hh pathway activation in thymic γδ T-cells modulated expression of genes and pathways which are involved in TCR signal transduction (*Vav3, Nfkbia*) (55) or are transcriptional targets of the TCR (*Nr4a3, Nr4a1, Egr1, Egr2*) (45), the overall impact of Hh-mediated transcription was the promotion of γδ T-cell development and maturation from the earliest CD25+CD73- stages, independently of any influence on TCR signal strength.

Signaling through the γδTCR also influences γδ T-cell differentiation after divergence from the αβ lineage: γδT17 development is believed to occur in the absence of TCR ligation, but γδTCR signaling is required for both γδT1 and γδNKT populations, and attenuation of TCR-signaling leads to expansion of γδNKT cells, suggesting that strong γδTCR signals favor differentiation of the γδT1 population (7, 53, 56, 57). Therefore, the impact of Hedgehog pathway activation on TCR signal strength may provide one explanation for the increase in γδNKT cells observed in the Gli2ΔN2 thymus; and the increase in *Ahr, Hes1*, and *Heyl* expression in the Gli2ΔC2 thymic γδ cells, as these genes are associated with the γδT1 population. Interestingly, the RNA sequencing analysis identified *Klf2* as a gene that is downregulated in CD27+CD3+γδ+ cells when Hh-mediated transcription is increased. Klf2 is required for efficient thymic egress and its deficiency increased the peripheral γδNKT population, many of which expressed CD4 (49). Consistent with this, we showed that Hh-mediated transcription also promoted the γδNKT population, and *Cd4* was upregulated in the Gli2ΔN2 CD27+CD3+γδ+ RNAseq datasets. In contrast, Shh-mutation reduced the thymic γδNKT population.

Short-term systemic inhibition of Hh pathway activation by *in vivo* pharmacological treatment with a Smo-inhibitor confirmed that Hh signaling is required for development and homeostasis of the γδ T-cell and γδNKT populations in the thymus, but Smoothened-inhibition also changed the splenic γδ T-cell subsets over the 2-week treatment period, decreasing the γδNKT population. This suggests that Hh signaling continues to actively maintain homeostasis of the splenic γδ populations, as well as influencing their development in the thymus. Given that the RNA sequencing analysis detected changes in the expression of genes that coordinate lymphocyte migration, it would be interesting in the future to assess the role of Hh signaling in γδ T-cell tissue localization and migration.

Hh pathway activation has been shown to limit αβ T-cell activation and Th1 differentiation, suggesting that Hh protein secretion by tumors may therefore be a mechanism by which tumors evade the adaptive immune response (23, 33, 44, 47, 58). In several studies Hh inhibition has been shown to increase αβ T-cell mediated immunity to cancer, suggesting that Hh-inhibitors may be used to enhance the immune response in immune-therapy (59–62). However, in our present study, we show that Smo-inhibition reduces the γδ T-cell and γδNKT populations in spleen and thymus, suggesting that Hh-inhibitors may not be suitable for use in conjunction with iNKT- or γδ cell-based immune-therapies, consistent with a previous report (63).

In summary, we show that Shh and Hh pathway activation in γδ T-cells are determinants of γδ T-cell development, maturation and effector subset distribution in the thymus, influencing multiple stages of development, and that Hh signaling continues to influence γδ T-cell populations in the spleen.

## Ethics Statement

Animal work was ethically reviewed at UCL, according to UK government regulations.

## Author Contributions

KM, C-IL, and TC conceived and designed experiments, analyzed data, and wrote the paper. KM, AS, MO, and JR advised on and analyzed RNA sequencing datasets. KM, C-IL, SN, SO, and SR performed experiments and analyzed data. SN, SO, MO, and JR critically reviewed the manuscript.

### Conflict of Interest Statement

The authors declare that the research was conducted in the absence of any commercial or financial relationships that could be construed as a potential conflict of interest.

## Supplementary Material

Supplementary Table 1 Shows DEG determined by EBayes statistics from the RNA sequencing datasets from thymic CD3+γδ+CD27+ cells between WT and Gli2ΔC2-tg.

Supplementary Table 2 Shows DEG determined by EBayes statistics from the RNA sequencing datasets from thymic CD3+γδ+CD27+ cells between WT and Gli2ΔN2-tg.
