# Supplementary material for: Sonic Hedgehog Is a Determinant of γδ T-Cell Differentiation in the Thymus
Source: Front Immunol. 2019 Jul 19;10:1629. doi: 10.3389/fimmu.2019.01629 (PMC6658896; doi:10.3389/fimmu.2019.01629)
Supplement: Supplementary Table 1 — Shows DEG determined by EBayes statistics from the RNA sequencing datasets from thymic CD3+γδ+CD27+ cells between WT and Gli2ΔC2-tg. [file Table_1.DOCX]

Supplementary Table 1

A list of 820 differentially expressed genes between WT and Gli2ΔC2-tg. Genes shown in blue were down-regulated in Gli2ΔC2-tg compared to WT, and genes shown in red were up-regulated.

| Cope | Sesn2 | Ccdc38 | Nrn1l | Mfn2 |
| --- | --- | --- | --- | --- |
| 2410006H16Rik | Mira | Zfp781 | Rab42 | Urod |
| Mgst2 | D930016D06Rik | Atp6v1g3 | Tnfrsf11a | Gpd1l |
| Pigx | Sept4 | Gm7854 | 4933430H16Rik | A930005H10Rik |
| Dok2 | Ing2 | Ralyl | Kcnip4 | Slc30a6 |
| Ifi27 | Ccdc50 | Ntf5 | Gm13154 | Pmpcb |
| Smdt1 | Dynlt1f | Srgap3 | 1700125H20Rik | Mettl13 |
| 2300009A05Rik | Utp23 | Tmem44 | Dact1 | Gins3 |
| Wbp1 | Tcf20 | Rfx8 | Ccdc39 | Rad18 |
| 1500012F01Rik | Rbm33 | Fam187b | 1700022H01Rik | Papd4 |
| Rab19 | Tet3 | Fam131a | Tspo2 | Chd1l |
| Vgll4 | Gamt | Asic1 | Alpl | Snord19 |
| Scarna2 | Mss51 | Cdh23 | Gm19395 | Nup153 |
| Commd6 | B4galt4 | Rasd1 | Lix1l | Leo1 |
| Fkbp2 | Rras | Crtac1 | Gm10433 | Hook1 |
| Arhgap30 | Derl3 | Brsk1 | Actl10 | Pfkl |
| Atp8b4 | Cdc37l1 | Arhgef17 | Gm12185 | Top1 |
| Dctn6 | S100a2 | Pcdhga5 | Bdh2 | Sars |
| Stk25 | E2f5 | Cyp2j6 | Ccdc78 | Abtb2 |
| Trim26 | Car15 | Cth | Nxf2 | Mad2l1bp |
| Fam195b | Lhfpl1 | Kctd12b | Angptl7 | Arhgap11a |
| Tor2a | Lrrc51 | 5330417C22Rik | Slc45a3 | Nedd1 |
| Idh3g | Gpr137b-ps | Abcb4 | Ttc12 | Srsf4 |
| Sdf2 | Cd209a | Sfrp5 | Tcea3 | Ddx18 |
| Nprl2 | Tsc2 | 4930524B15Rik | Asap2 | Topors |
| Mea1 | Atm | Jam3 | Srp54a | Rrn3 |
| Zcchc7 | Icam4 | Ryr1 | Myo10 | Vrk2 |
| Lancl1 | Rhod | Efna2 | H2afy2 | Nsf |
| 1110007C09Rik | Ddhd1 | Adhfe1 | Klf8 | Bms1 |
| Spsb3 | 9930104L06Rik | A4galt | Crispld2 | Cox18 |
| Ly6g5b | 4930478L05Rik | Ttc39a | Hoxa3 | Mrpl45 |
| Slc25a45 | 1110034G24Rik | Adam6b | Arfgef2 | Mir7024 |
| Naga | Mlf1 | Tnfsf15 | C230035I16Rik | Iscu |
| Dedd2 | Alpk1 | 2610027K06Rik | Far1os | Prdm4 |
| Prrc2c | Ccdc176 | Trpc3 | Asb2 | Pcmt1 |
| Mau2 | Lgmn | Ksr2 | Hspa2 | Prpsap2 |
| Meis3 | Bbs2 | Peg12 | Sipa1l3 | D6Wsu163e |
| Tspan32 | Slc19a2 | Gm4951 | Intu | Nadk |
| Tsr3 | Dlg1 | Mest | Fxyd4 | Mars |
| 5830432E09Rik | Med13l | 9330182L06Rik | Pnldc1 | Trappc11 |
| Aip | Kctd15 | Mycbpap | Ninj2 | Ppp1r8 |
| Galnt6 | Ocrl | Cav2 | Fsip1 | Fam65b |
| Zfp935 | Dock9 | Mroh8 | Pilrb1 | Snord55 |
| Rbm10 | Mkln1os | Pkd1l2 | Gm15326 | Cblb |
| Dnajc5 | Rnf185 | Masp2 | Samd11 | Exoc3 |
| Lactb2 | 4930402H24Rik | Ugt1a1 | Asb5 | Ckap2 |
| Kcmf1 | Cmah | Slc6a20b | Nsun6 | Cenpq |
| Apbb1ip | Pspn | Bhlhe41 | Tgm3 | Rpf1 |
| Pld4 | 4632404H12Rik | Fbn2 | Nudt15 | Mrpl16 |
| Snord35b | Tbc1d8b | Jph3 | Selm | Cpsf2 |
| Sltm | Rbm4 | Unc45b | Abca3 | Pex2 |
| Smim4 | Prox2 | Abcg4 | Ankib1 | Cdc6 |
| Tmem120b | Paqr7 | Adamts4 | Polr3g | Mad1l1 |
| Pisd-ps2 | Ptges3l | Cep112 | Hsd17b7 | Aasdhppt |
| Icam1 | Tatdn2 | Cnga3 | Macrod2 | Ly6k |
| Trip11 | Armc3 | Zan | Fam71b | Hccs |
| Alg5 | Slc27a2 | Rbfox2 | Riok2 | Elavl1 |
| Wdr83 | Ino80 | Cps1 | Tma16 | Ifit1 |
| Podnl1 | Mblac2 | Phldb2 | Abhd2 | Epb4.1l2 |
| Traf3ip2 | Cxcl17 | Arnt2 | Tbc1d22bos | Ddb1 |
| Ndufaf1 | Ncald | Grin3a | Phc1 | Pus1 |
| Mms19 | Tnks | Tenm4 | Chga | Nsun2 |
| Mgmt | Lrrc29 | Eppk1 | Sdr42e1 | Hmgb3 |
| Rabgap1l | Peg13 | Ttbk1 | 1810034E14Rik | Ide |
| Cox11 | Wscd1 | Muc2 | Angptl6 | Ddx24 |
| Thap7 | Degs2 | Prrt3 | Jrk | Thoc3 |
| Kif21b | Tex38 | Acan | 2010109A12Rik | Nolc1 |
| Gstt2 | Phldb3 | Syt6 | Oscp1 | Tmem71 |
| Klc4 | Zfp467 | Lamc2 | Cd276 | D19Bwg1357e |
| Btbd6 | Ralgapa2 | Fam160a1 | Gcnt7 | Tdp1 |
| Exoc7 | Marf1 | Ifnlr1 | Uprt | Mphosph6 |
| Mppe1 | Pfkfb1 | Lrrc8e | Zfp786 | Aatf |
| Timm44 | Serpina3f | C5ar2 | Prr11 | Wdr36 |
| S100a13 | Errfi1 | Ctcfl | Slc35a3 | Hexb |
| Dnajc4 | Trim46 | A230056J06Rik | Sult2b1 | Rqcd1 |
| 2810408A11Rik | Erc1 | Tenm1 | 0610010F05Rik | Taf6 |
| Gps1 | Lrba | Rin1 | Arhgap10 | Mir8119 |
| AA465934 | Blnk | Fam65c | Saal1 | Med14 |
| Ccdc101 | Selenbp1 | Esrp1 | Tns1 | Mrpl46 |
| Gtpbp6 | Ino80d | Cyp1a1 | Phtf2 | Exosc10 |
| Klhl20 | Tnfsf9 | Ddn | Cyp4f41-ps | Seh1l |
| Metap1 | Rsg1 | Ephx2 | Zcchc2 | 9430016H08Rik |
| Zfp707 | Adam15 | Nox1 | Dnajc16 | Naa50 |
| Zfp407 | Dmxl1 | Slc22a8 | Slpi | Umps |
| Spns3 | 9330020H09Rik | Vmn2r85 | Tdrd3 | Emc4 |
| Rgs18 | Tsc1 | Lrrc63 | Map1lc3a | Mrps22 |
| Klhl2 | Znrf3 | Fbxo40 | Abt1 | Gli2 |
| BC025920 | Gm19557 | Cks1brt | Trmt10a | Nup93 |
| Pisd | Gm16617 | B430010I23Rik | Helq | Sarnp |
| Arhgef4 | Dbp | Gja5 | Ap1s2 | Mtap |
| Tex264 | Tmcc3 | Pcdhga2 | Dtd2 | Ctcf |
| Lyrm4 | Rin2 | Sox30 | 5730405O15Rik | 2700062C07Rik |
| Tmem206 | Clec1b | Eva1c | Rbm3os | Sec23b |
| Stk39 | Fbxo4 | Megf8 | Lrp8 | Acsl5 |
| Cds2 | 1700086O06Rik | Fndc8 | Lrrc8d | Syce2 |
| Pigv | Carf | Reep6 | Rbms2 | Brd7 |
| Nlrc3 | Timmdc1 | Ppp2r2c | Pak1 | Hadh |
| Fam118b | Zfp341 | A830010M20Rik | Tada2a | Oat |
| Snord89 | Cd80 | Grb14 | Lgals7 | Me2 |
| Lsm14b | Spns2 | Hpdl | Vav3 | Nt5dc2 |
| Mir8113 | Ece2 | Lrrn4cl | Clic3 | Cse1l |
| Ercc3 | 4930473A02Rik | Fam227b | Fam104a | Fh1 |
| Ip6k2 | Gm16386 | Srms | Lsg1 | Trap1 |
| Rtn4 | Zc3h6 | 1700123J17Rik | Cebpz | Rars |
| Isoc2a | 2010107G23Rik | Asprv1 | Rpap2 | Fkbp5 |
| AI987944 | Ccdc63 | Trim72 | Snora30 | Nucks1 |
| Stoml1 | Clec10a | Nbl1 | Sass6 | Mthfd1 |
| Zfp932 | Stc2 | Sostdc1 | Ptar1 | Rad21 |
| 1110065P20Rik | AA415398 | Fsd1 | Fam175b | Psip1 |
| Nat9 | Gm2518 | Egr4 | Parp16 | Nup37 |
| Chka | Npm2 | Fcrl5 | Cebpg | Gart |
| Fam134b | Rab5b | Unc13a | Mtrr | Ruvbl2 |
| Milr1 | Jazf1 | Rufy4 | Zik1 | Dkc1 |
| H2-Q4 | Hspa1a | Esr2 | Itga6 | Dtl |
| Pfkfb3 | Adam19 | Lrrc3b | Gpt | Rab10 |
| Msi2 | Tbc1d12 | Lmod3 | Rab6b | Sfr1 |
| A330069E16Rik | Ramp2 | Nlrp6 | Ncoa2 | Nars |
| Bod1l | Syp | Gphb5 | Adar | Smarca5 |
| Usp34 | Zglp1 | Omp | Snord16a | Nap1l4 |
| 4933434E20Rik | Fam81a | Insm1 | Mcm8 | Tbrg1 |
| Tpm1 | Mkrn3 | Habp2 | Recql | Isy1 |
| Tatdn3 | Rnf43 | Krt85 | Dync1i2 | Rbm7 |
| 9330133O14Rik | Agap2 | Plcd1 | Psrc1 | Mir7115 |
| Pigl | Zmynd8 | Psma8 | Bbs9 | Dnajc8 |
| Baz2a | AI839979 | Gsto2 | Armc1 | Serbp1 |
| Golt1b | Zdhhc23 | Serpina1e | Tmem205 | Gars |
| Qpctl | Bex4 | Akr1c6 | Cars | Snx3 |
| Eid2 | Zfp773 | Lrrc34 | Wbscr27 | Snrnp40 |
| Zfp882 | Astl | Vldlr | Aplp2 | Erh |
| Fam174b | Zfp449 | Steap4 | Snord92 | Mrfap1 |
| Map3k8 | Mocos | Mnda | Hspb6 | Ddx1 |
| Chd7 | Gab2 | Lrrc25 | Hyou1 | Lta4h |
| 3110062M04Rik | AI182371 | Sgca | Rab32 | Fkbp4 |
| Tmem87a | 1700026D11Rik | Mtus1 | Chd3os | Srsf3 |
| Eri3 | Cdc42ep2 | Ccdc180 | Gle1 | Sae1 |
| Fut11 | Acta2 | Paqr6 | Retsat | Phgdh |
| Zdhhc1 | Anks6 | Cntn1 | Zbtb8a | Rgcc |
| 2410131K14Rik | Rfxank | Scnn1a | Slc10a3 | Paics |
| Cish | Tbc1d2 | Cthrc1 | Heatr1 | Snrpa1 |
| Hspa1b | Trnp1 | C030029H02Rik | Mir3091 | Psme3 |
| Maged1 | Timm8a2 | Gm4961 | 3830403N18Rik | Pdia6 |
| Slc35g1 | Adcy9 | Neurl1a | Rab28 | Cdc20 |
| Gm10033 | Apon | Jsrp1 | Kctd13 | Ywhab |
| Thap4 | D930048N14Rik | 6030468B19Rik | Sgk1 | Sf3a3 |
| Osbpl3 | Nrep | Aif1l | Dhx32 | Psmd12 |
| Dpagt1 | BC037704 | Bmp8a | Gmds | Mcm4 |
| Cdip1 | Arhgef5 | D330045A20Rik | Ahr | Ncl |
| Arsa | Adamts14 | C430049B03Rik | Mxd1 | Tubb4b |
| Il12a | Mrgpra9 | H2-M10.2 | Tbccd1 | Eif3d |
| Pcyt1a | Mmp19 | Amt | Urb2 | Calr |
| Ankrd12 | Tmem239 | 0610039K10Rik | Lrrc14 | Npm1 |
| Pik3r5 | Kbtbd12 | Pllp | Sergef | Phb2 |
| Cd44 | Znf41-ps | Arhgef9 | Lancl2 | Cdca7 |
| Slc25a19 | Hnf1a | A930002C04Rik | Btd | Ddx39 |
| Arl8a | Pla1a | Ly6i | Mir324 | Stip1 |
| Fgfrl1 | 1700112J16Rik | Gm19276 | Msl3 | Ccnb2 |
| Fam193a | C1ql1 | H2-M5 | Snrpa | Tcp1 |
| Atcayos | Slc10a5 | Hoxaas3 | Phax | Lsm3 |
| Gstt3 | Itga2b | Pcbd1 | Ttc7b | Rbbp7 |
| Fbxl15 | Fam78b | B4galnt2 | Pop4 | Slbp |
| A930024E05Rik | Pianp | Foxp3 | Cnep1r1 | Cct6a |
| 1700102H20Rik | Tmprss9 | Arrdc5 | Kat5 | Mcm6 |
| Zkscan8 | Fndc3b | Acot10 | Txndc9 | Rps27l |
| Nicn1 | Cd22 | Kdf1 | Trim27 | Hnrnpu |
