# Supplementary material for: Sonic Hedgehog Is a Determinant of γδ T-Cell Differentiation in the Thymus
Source: Front Immunol. 2019 Jul 19;10:1629. doi: 10.3389/fimmu.2019.01629 (PMC6658896; doi:10.3389/fimmu.2019.01629)
Supplement: Supplementary Table 2 — Shows DEG determined by EBayes statistics from the RNA sequencing datasets from thymic CD3+γδ+CD27+ cells between WT and Gli2ΔN2-tg. [file Table_2.DOCX]

Supplementary Table 2

A list of 1218 differentially expressed genes between WT and Gli2ΔN2-tg. Genes shown in blue were down-regulated in Gli2ΔN2-tg compared to WT, and genes shown in red were up-regulated.

| Rsrp1 | Fam63b | Trnp1 | 4732471J01Rik | Stam2 |
| --- | --- | --- | --- | --- |
| Pdcd1 | Dnajc4 | Arhgap20 | BC020402 | Cox18 |
| Anxa2 | Nat9 | Pomc | Rnf144b | Akr1e1 |
| Arf5 | Ing2 | Anxa9 | Tmcc3 | Naa50 |
| Kcnn4 | Necap2 | Gm7694 | Ckm | Dus4l |
| Flna | Snord89 | Lrrtm1 | Hs3st1 | Rnu73b |
| Abhd8 | Sfn | Clec10a | Colgalt2 | 3110009E18Rik |
| Klf2 | Siah2 | 4931431C16Rik | Hmgb1-rs17 | Spryd7 |
| Nr4a1 | Strada | Sytl4 | Phf11a | Ppip5k2 |
| Rasa3 | Smurf1 | Erg | Thbs1 | Scaf11 |
| Arf1 | Wdr34 | Pid1 | Ccnd1 | Mrps31 |
| Ptbp1 | Dusp4 | Lrba | Cyp4f18 | Ube4a |
| Cyba | Zdhhc1 | Rbm12b1 | Ubald2 | Wdr44 |
| Tcf3 | Srf | Lif | Lix1l | Zfp938 |
| Sytl1 | Slc35f6 | Gm13483 | Pou6f1 | Mr1 |
| Hnrnpab | Ccrn4l | Pvrl2 | Kif1c | Cebpz |
| Anapc5 | Jrkl | Asic1 | Ptgdr2 | Zp1 |
| Vgll4 | Scaf1 | Crabp2 | Stx19 | Ddx18 |
| Rn45s | Tef | Kirrel3 | Mei1 | Gadd45a |
| Gramd4 | Hk1os | Dbx2 | Pih1d2 | Pex16 |
| Cdc37 | Plekhm2 | Nrep | C330013J21Rik | Rabgap1l |
| Pycr2 | 2410131K14Rik | Fdxacb1 | Adam12 | A630023P12Rik |
| Dok2 | Rhobtb2 | Gpc3 | 9430065F17Rik | Zcchc11 |
| Lypla2 | 1700094D03Rik | Siglecg | Gm14325 | Nol11 |
| Uhrf1 | Gmeb2 | Mir684-1 | Hemgn | Rnf146 |
| Tomm40 | Hspa1b | Fzd7 | Zmym4 | Nedd1 |
| H2-Ab1 | Sgsh | BC037704 | Atf3 | Ccdc55 |
| Cdkn2d | Jag2 | Adamts14 | Zfp658 | Trappc2 |
| Fam168b | Nmrk1 | Hnf1a | Tmem26 | Arl4a |
| Cd1d1 | Dis3l | Prss23 | Ccl24 | Rexo4 |
| Emid1 | Acat2 | Astl | Rasd1 | Pknox1 |
| BC017643 | Slc25a19 | Adcy9 | Nudt15 | Eci1 |
| Cnppd1 | Thumpd2 | Pla1a | Gm10638 | Rab14 |
| Por | Msl3l2 | Htr2b | Gm9895 | Pmepa1 |
| Ubxn1 | Fastkd5 | C1ql1 | Adap2 | Trmt11 |
| Tor2a | Nacc1 | H2afy2 | Pik3cb | Zfp943 |
| Grap | Pla2g6 | Tmprss9 | Zhx2 | Rnase4 |
| Pxn | Baz2a | Svip | Atad3aos | Zfpl1 |
| Prkcsh | Ciapin1 | Ak4 | Fam89a | Tmx2 |
| Dedd2 | Fbxl15 | Atf5 | C030037D09Rik | Fam204a |
| Sh2b1 | Gm15706 | Abcb4 | Alg6 | Nosip |
| Med24 | Dennd1a | Zfp781 | Pilrb1 | Zfp143 |
| Snord35b | Rnf157 | Hpx | Tom1 | Hars |
| Adck5 | Aen | 1700112J16Rik | Espn | Orc4 |
| Matk | Tmem180 | Vmn2r95 | 1700093K21Rik | Prelid2 |
| Wbp1 | Il11ra1 | Elfn2 | Gm20735 | Esco1 |
| Man1a | Zfp935 | Crtac1 | Prss34 | Sec31a |
| Ddit4 | Glb1l | Pcdhga5 | Hsd17b7 | Gpcpd1 |
| Ccar2 | Rad9b | Them7 | Prmt10 | Egln1 |
| Clk3 | Pias3 | T2 | Ly6i | Arntl |
| Cpt1a | Zfp235 | Mnd1-ps | 4632415L05Rik | Ccnc |
| Sptbn1 | Pigh | Bpi | Hpgds | Zmym2 |
| Arl6ip4 | Wdr5 | Cyp2d26 | Echdc1 | Atg5 |
| Cdk2 | Rnf216 | Adamts7 | Yy2 | Tor1aip1 |
| Usp5 | Snx30 | Fam78b | Gkap1 | Dtd1 |
| Tssc1 | Gpatch3 | 4933408B17Rik | Zfp715 | Armc1 |
| Sigirr | Acsf3 | Gm6194 | N4bp2l2 | Zfp120 |
| Gtpbp1 | Cyb561a3 | 1700061G19Rik | Rasl11a | Ppil4 |
| Tmem120b | Rhbdl1 | Adamts20 | Ttyh2 | Alg5 |
| Ecm1 | Lnx2 | Cth | Lnp | Isg20 |
| Wfs1 | Anxa1 | Cpeb1 | Mkl2 | Cebpzos |
| Arhgef2 | Socs5 | Siglece | Stk38l | Trim21 |
| Otulin | Ern1 | Sfrp5 | C230091D08Rik | Pdk3 |
| Tab1 | Man2a1 | Tle1 | Abhd2 | Stk26 |
| Mmd | Mlst8 | Fcrl6 | Rpusd2 | Uba5 |
| Acaa1a | Selenbp1 | Alox8 | N4bp1 | Pttg1 |
| Slc25a38 | Cds2 | Ksr2 | Kansl2 | Stambp |
| C2cd5 | A330069E16Rik | Ryr1 | Arhgdig | Tpr |
| Ap1s1 | Atxn7l3 | Phf21b | Csrp2 | Idnk |
| Men1 | Zfp91 | Fam131a | Sco1 | Slu7 |
| Galk1 | Tet3 | 4930565N06Rik | Ogfod1 | Aimp2 |
| Tle3 | 9330133O14Rik | Gli1 | Mllt4 | Slc3a2 |
| Arap1 | Pcyt1a | Gpr35 | Myo5b | BC004004 |
| Usp7 | Lrrc29 | 2610027K06Rik | Zfp772 | Sars |
| Slc25a1 | Hist1h4k | A330093E20Rik | Atp2b4 | Psme2b |
| Pagr1a | Arfip2 | Kalrn | Rnf113a1 | Tsta3 |
| Rcor1 | Ankrd40 | Hecw2 | Ptprn2 | Brd7 |
| Zer1 | Ubr5 | Acsbg2 | Bbs9 | Fabp4 |
| Nt5dc2 | Fads2 | F630028O10Rik | Maml2 | Irf8 |
| Metrn | Mss51 | Dnaaf1 | Tubg2 | Wdr83os |
| Atf6b | Cmah | Trpm2 | Wnt4 | Mkln1 |
| Aim2 | Gstt3 | Lamb1 | Luzp1 | Nolc1 |
| Spsb3 | Prr14l | Sema3c | Mmp14 | Zfp280d |
| 1700017B05Rik | Nfe2 | Fam131b | Zik1 | Nabp2 |
| Ankrd16 | Tex9 | Elovl4 | Mir1b | Papolg |
| Mrpl24 | Il20ra | Pkd1l2 | Dhx32 | 1810041H14Rik |
| Rapgef3 | Klc3 | Mroh8 | Hspb6 | Icos |
| Kxd1 | Gemin5 | Tmem253 | Mios | Aftph |
| Cd200 | Fam102b | Masp2 | Gm10640 | Commd8 |
| Tmem63a | Fgfrl1 | Ugt1a1 | Pde5a | Tada2a |
| Rnf10 | Fbxw7 | Fermt2 | Slc25a32 | Rbm6 |
| Rnu11 | Bbs2 | Ccdc74a | Rps6kb1 | Pdcd2l |
| Fahd2a | Pim3 | Fam179a | 4833412C05Rik | D16Ertd472e |
| Cpq | Gcnt4 | Adamts4 | Pcbp3 | Lrrc17 |
| Get4 | Rnf19a | Gm21992 | Gna15 | Aatf |
| Klc4 | Atcayos | Cnga3 | Retsat | Ccdc43 |
| Ccne1 | Lrrc14 | Hrh1 | Txlng | Batf3 |
| Abcb8 | Mid1 | Vegfc | Mospd2 | Mrps22 |
| Mboat7 | Tceal3 | Cct8l1 | Olfml3 | Exoc3 |
| Kcmf1 | Hist1h2ah | Slit1 | Tctex1d1 | Mrpl39 |
| Pbx4 | Id1 | Apc2 | Phlda3 | Mcl1 |
| Sirt3 | Zfp764 | Pde9a | 1700120C14Rik | Gars |
| H2-Ob | Irgq | Efna2 | Mxi1 | Papd4 |
| Slc35b2 | Il12a | Chd5 | Snrnp35 | Rgs2 |
| Tor1a | Nr4a3 | Col14a1 | Ankrd52 | Tubb2a |
| Prkab1 | Armcx6 | Otogl | Peak1 | U2surp |
| Etv3 | 9230110C19Rik | Unc45b | St6galnac3 | Higd1a |
| Rcc1 | Timm44 | Fam184b | Top1mt | Odc1 |
| Adora2a | Akap1 | Enah | Sorbs1 | Ncoa7 |
| Rnf135 | LOC106740 | Dnah2 | Ctns | Rngtt |
| Ilk | Zfp275 | Slc1a2 | Fam83a | Slc25a39 |
| Slc4a2 | Smurf2 | Hecw1 | Acyp2 | Eprs |
| Zfand2b | Ppm1d | Amotl1 | 4930549G23Rik | Atp6v1b2 |
| Nprl2 | 1110034G24Rik | Kcnn3 | AI429214 | 4930453N24Rik |
| Pik3r2 | Bcam | Col25a1 | Kbtbd3 | Epcam |
| Wdr46 | Csrnp2 | Tecta | Gzmm | Taok3 |
| Meis3 | Zbed3 | Kcnt1 | Ppp1r2 | 0610037L13Rik |
| Bag6 | Lhfpl1 | Aqp4 | Mrgpra1 | Pcsk1 |
| Psmd9 | 4931406P16Rik | C4b | Hspa13 | Rars |
| Fbxw8 | Rbm33 | Kl | Tnnt1 | Ccdc101 |
| Metap1 | D830046C22Rik | Abcc8 | Pes1 | Cept1 |
| Ppt2 | Cers6 | Nr2f2 | Arfgap3 | Pias1 |
| Carhsp1 | Nipsnap1 | Ahrr | Zfp808 | Samhd1 |
| Tmed1 | Mfap3 | Adgrg2 | Dhodh | Prr29 |
| Anks1 | Pfkfb1 | Snap91 | Nebl | Tcea1 |
| Eng | D130040H23Rik | Slco5a1 | Fxyd4 | Pcmt1 |
| Kti12 | Usp35 | Pdzrn3 | Nckap1 | Ccz1 |
| Cd72 | Mrs2 | Cald1 | Wdr75 | Ube3a |
| Ccdc9 | Derl3 | Cd36 | Mir6399 | Usp15 |
| Wsb2 | Adam15 | Prlr | Tctex1d2 | AI413582 |
| Stoml1 | Pcgf3 | Ccdc158 | Cltb | Hnrnpf |
| Zfp445 | Rad51d | Miat | Dcun1d4 | Ubc |
| Zbtb17 | Sv2a | Naip6 | Arl13b | Cse1l |
| Rab4a | Zscan10 | Vcan | Tdrd3 | Ndufs1 |
| Spns3 | Med20 | Nxf3 | Ssx2ip | Spsb1 |
| Hadha | Reep1 | Fam65c | Btbd9 | Cpe |
| Ddx19a | Pcyox1l | Creb5 | Iigp1 | Ptcd3 |
| Cbx4 | Ccl25 | Pde6b | Slc35a5 | Eif3a |
| Nit1 | Kif3c | Saxo2 | Zfp846 | Tmem9b |
| Coq2 | Sort1 | C1ra | Gcc2 | Azin1 |
| Dennd3 | Ovgp1 | Kcns1 | Rit1 | Mtap |
| Yipf2 | Fam110b | Cbln2 | Sostdc1 | Mphosph6 |
| Josd1 | BC051537 | Ephx2 | Acaca | Laptm4a |
| Scin | Rps6ka2 | Lilra6 | Klhdc1 | Tspan3 |
| Ralgds | Fgfbp3 | Mcmdc2 | Rbmx2 | Nmd3 |
| Kctd5 | Rras | Srms | Mycbp2 | N4bp3 |
| Dapl1 | Elk1 | Tspan12 | Mfn1 | Nabp1 |
| Mon1a | 4930520O04Rik | Fn1 | Gpatch4 | Dnttip2 |
| Mettl8 | Pspn | P4ha3 | Alad | Ddx1 |
| B3gnt2 | Mapk12 | Hpcal4 | Plxnd1 | Trap1 |
| Traf7 | Bmp2k | Ccser1 | Dnajc1 | Erp44 |
| Alkbh1 | Prox2 | Gm4961 | Cd84 | Atraid |
| Man2c1 | Tatdn2 | P3h2 | Fam175b | Cnot2 |
| Esrra | Det1 | F11 | Pggt1b | Ngdn |
| Taf1c | Trim46 | Tenm4 | Cpped1 | Nars |
| Iffo1 | Rsg1 | Hoxb4 | Arel1 | Fdps |
| Osm | Pelp1 | Hsf5 | 3010026O09Rik | Sesn1 |
| Dnaja3 | Spag1 | Daam2 | Sgk3 | Iscu |
| Ivd | Glyctk | Gm10433 | Socs2 | Aprt |
| Exoc7 | 6030458C11Rik | Synpo2l | Slc10a3 | Gm11346 |
| Slc15a2 | Irak3 | Gm6756 | Tmem69 | Fkbp5 |
| Trp53rka | Tnfrsf13c | Dlgap1 | Ptar1 | Mrps34 |
| Ankrd39 | Trmt44 | Ankar | Dhx40 | Me2 |
| Tsr3 | Gse1 | Oscar | Zfp518a | Xrn2 |
| Snora78 | Wscd1 | Pawr | Csnk1g3 | Ddx24 |
| Pbx2 | Apol9b | Cfap45 | Rad52 | Lmnb1 |
| Slc2a1 | Disp1 | Gm14092 | Atg14 | Gpr183 |
| Wls | Wdpcp | 4933400F21Rik | Zfp961 | Far1 |
| Arvcf | Fancd2os | Nbl1 | Tcerg1 | Mrpl46 |
| 2810408A11Rik | Ankrd24 | Spata17 | Ascl1 | Slc27a2 |
| B230219D22Rik | Spry4 | 4931408D14Rik | Npepps | Hspa9 |
| Snapin | Qrfp | Serpina10 | Ripk2 | Ddx3x |
| Trmt1l | Gjb2 | Slc38a11 | Wdr92 | Dnajc3 |
| Dedd | Cd209d | 4930538K18Rik | Klf7 | Gpbp1 |
| Vps39 | Ccdc114 | Vldlr | Mcpt8 | Fundc1 |
| Zfp362 | Znrf3 | Zfp185 | Smim3 | Slfn1 |
| Gprin3 | Nrp2 | Ecel1 | Nampt | Uqcc3 |
| Cyhr1 | 4632404H12Rik | Teddm1a | Ap2b1 | Nfkbia |
| Agpat3 | Dbil5 | Gm572 | Snord58b | Ywhah |
| Adgrg3 | Adgra2 | Dio3os | Iqcb1 | Creld2 |
| Zfp553 | Tha1 | Styxl1 | Derl2 | Eif4a2 |
| Ggact | Lipe | Nlrp6 | Riok2 | Bag1 |
| Arih2 | Gipc3 | Gm7904 | Tdrd5 | Bola1 |
| Ccdc166 | Msh5 | Actn3 | Slc17a5 | Dld |
| Akr1b10 | Mmp17 | SlcUbl4a | Bcl2a1a | Papola |
| Agtrap | Mst1 | Gm5803 | Lman1 | Ywhaq |
| Kif21b | Art2a-ps | Sco2 | Trnt1 | Dnajb11 |
| Klhl2 | Slc9b2 | Pabpn1l | Lipo1 | Klk8 |
| Ska2 | Pmel | Gm960 | Arf6 | U2af1 |
| St3gal5 | Gm16617 | 6720483E21Rik | Atp11c | Epsti1 |
| Ccdc61 | Syde1 | Iltifb | Ptcd2 | Serbp1 |
| 1110032F04Rik | Gm16907 | Tyms-ps | Dcp1b | Fkbp4 |
| Mpg | 2900079G21Rik | Rin1 | Lsg1 | St3gal6 |
| Tlr1 | Axin2 | Ucp3 | Gtf2a1 | Tra2b |
| Lpin2 | Proz | Mical3 | Crls1 | Eif5 |
| Ints7 | Tnfsf9 | Elmod1 | Cpsf2 | Prps2 |
| Cdipt | Car15 | Ophn1 | Eif4e2 | Btg1 |
| Zfp213 | Hdac9 | Scnn1a | Prdm4 | Jakmip1 |
| Tmco6 | 5031439G07Rik | Mgll | Cluap1 | Rab8a |
| Rfwd3 | Tex38 | E330011O21Rik | Rnf6 | Junb |
| Rgs18 | Nxn | Fam167b | S100a1 | Eif3d |
| Zdhhc8 | Zfp438 | 1700016K19Rik | Slc16a6 | Psme3 |
| Zmym3 | Cabyr | Slit3 | Ankrd49 | Sri |
| Ndrg2 | Meis1 | Dram1 | Fam172a | Ech1 |
| Rrp12 | 1700008J07Rik | Grm6 | Lgmn | Hsph1 |
| Dnajc10 | Alpl | Cbs | Cnih4 | Isg15 |
| Ldlrap1 | S100a2 | Tnfsf18 | 4933412E12Rik | Tbrg1 |
| Sphk2 | Hs1bp3 | Zfp879 | Nsun3 | Akr1c13 |
| Plxdc2 | Spns2 | Angptl7 | D19Bwg1357e | Bst2 |
| Stard3 | 1700040L02Rik | 5730416F02Rik | Dnajb6 | Rtp4 |
| Coil | Cahm | Car5b | Snx10 | Manf |
| 5031425E22Rik | Nudt17 | Ccdc152 | Snord42a | Xbp1 |
| Gtpbp6 | Gm3219 | Arhgef9 | Nol8 | Snx2 |
| Zkscan6 | Map1lc3a | Slc30a3 | Ms4a6d | Prdx6 |
| Exoc3l | Ccdc63 | Txlnb | Ikzf5 | Stip1 |
| Rgs12 | Lrrc14b | A430090L17Rik | Gm11110 | Rmnd5b |
| Def8 | Arhgef5 | Kynu | Pklr | Sec11a |
| Ubiad1 | Upk1a | Ptch2 | Kctd12 | Mbnl1 |
| Zfp574 | Gadl1 | Prkce | Rsu1 | Ifi47 |
| Dclk2 | Gab2 | Fbxl13 | A430035B10Rik | Cct8 |
| Fam109a | Slc7a4 | Kcnc1 | Mad1l1 | Pdia4 |
| Urgcp | Ildr1 | Yes1 | Leo1 | Pdia6 |
| Brip1os | 4930473A02Rik | 9030204H09Rik | Gm10509 | Ckb |
| Atmin | Slc30a7 | Tmc5 | Gabpb1 | S100a11 |
| Ttc9c | Rasl2-9 | Neurl1a | Bik | Clec12a |
| Arsa | 4932416H05Rik | Kcnc3 | Wapal | Sept7 |
| Pqlc3 | Serinc4 | 1700003G18Rik | D6Wsu163e | Sepp1 |
| Snord83b | Hdc | Gm5111 | Mrps10 | Slfn2 |
| Ap4m1 | Cd80 | Shisa4 | Cnot10 | Fxyd7 |
| Pprc1 | Tbx19 | 1700034I23Rik | Mrpl19 | Rexo2 |
| Pex10 | Rhd | Fam163b | Rbbp9 | Ifi27l2a |
| Slc25a25 | Avpr1a | 4933406I18Rik | Cited4 | Calr |
| Spata2l | Ylpm1 | Pank1 | Ptpn2 | Cd8a |
| Fbxo33 | Ltc4s | Fam81a | Taf13 | Cdc42 |
| Galns | Klrb1 | A730085K08Rik | Msra | Hspa8 |
| Paqr7 | Rec114 | Tnfsf12 | Golph3l | Pdia3 |
| Slc35g1 | Tm4sf5 | Mt2 | Herc6 | Ltb |
| Tpm1 | Smkr-ps | Tmtc2 | Xrcc4 | Hspa5 |
| Stk19 | Echdc3 | Gpr137b | Gmds | Ly6e |
| Armcx2 | A430105I19Rik | Zfp69 | D1Ertd622e | Ms4a4b |
| Qpctl | Rasgrf2 | Mixl1 | Fam69a | Cd52 |
| Aff3 | Snx16 | Itga3 | Zfp68 | B2m |
| Vav2 | 4831440E17Rik | Adrb1 | Agfg2 |  |
| Gins2 | Tigd3 | Fn3k | Stam2 |  |
